# The Effect of Topo-Climate Variation on the Secondary Metabolism of Berries in White Grapevine Varieties (*Vitis vinifera*)

**DOI:** 10.3389/fpls.2022.847268

**Published:** 2022-03-08

**Authors:** Kelem Gashu, Chao Song, Arvind Kumar Dubey, Tania Acuña, Moshe Sagi, Nurit Agam, Amnon Bustan, Aaron Fait

**Affiliations:** ^1^Albert Katz International School for Desert Studies, Jacob Blaustein Institutes for Desert Research, Ben-Gurion University of the Negev, Beersheba, Israel; ^2^Albert Katz Department of Dryland Biotechnologies, French Associates Institute for Agriculture and Biotechnology of Drylands, Jacob Blaustein Institutes for Desert Research, Ben-Gurion University of the Negev, Beersheba, Israel; ^3^Wyler Department of Dryland Agriculture, French Associates Institute for Agriculture and Biotechnology of Drylands, Jacob Blaustein Institutes for Desert Research, Ben-Gurion University of the Negev, Beersheba, Israel; ^4^Ramat Negev Desert Agro-Research Center, Ramat Negev Works Ltd., Halutza, Israel

**Keywords:** phenylpropanoid metabolism, carotenoid degradation, metabolite profiling, oxidative stress, high temperature

## Abstract

Exploiting consistent differences in radiation and average air temperature between two experimental vineyards (Ramat Negev, RN and Mitzpe Ramon, MR), we examined the impact of climate variations on total carotenoids, redox status, and phenylpropanoid metabolism in the berries of 10 white wine grapevine (*Vitis vinifera*) cultivars across three consecutive seasons (2017–2019). The differences in carotenoid and phenylpropanoid contents between sites were seasonal and varietal dependent. However, the warmer RN site was generally associated with higher H_2_O_2_ levels and carotenoid degradation, and lower flavonol contents than the cooler MR site. Enhanced carotenoid degradation was positively correlated with radiation and daily degree days, leading to a greater drop in content from véraison to harvest in Colombard, Sauvignon Blanc, and Semillon berries. Analyses of berry H_2_O_2_ and phenylpropanoids suggested differences between cultivars in the links between H_2_O_2_ and flavonol contents. Generally, however, grapes with higher H_2_O_2_ content seem to have lower flavonol contents. Correlative network analyses revealed that phenylpropanoids at the warmer RN site are tightly linked to the radiation and temperature regimes during fruit ripening, indicating potentially harmful effect of warmer climates on berry quality. Specifically, flavan-3-ols were negatively correlated with radiation at RN. Principal component analysis showed that Muscat Blanc, Riesling, Semillon, and Sauvignon Blanc were the most site sensitive cultivars. Our results suggest that grapevine biodiversity is likely the key to withstand global warming hazards.

## Introduction

By the end of the 21st century, mean global air temperature is expected to rise between 1.5 and 2°C in most of the world’s wine-growing regions (Ullah et al., 2020). Consequently, the longstanding relationship between geography and viticulture will be disrupted, necessitating changes to the wine industry (Wolkovich et al., 2018). A recent study showed that a 2°C rise in air temperature could result in a 24–56% loss of the viticultural area within current wine-growing regions (Morales-Castilla et al., 2020), the consequences of which are yet unknown. As elevated temperature has been reported to affect fruit chemical compounds, the essential components of wine quality, such projected air temperature change may impact the production of high-quality wine. In light of this, a recent extensive study that assessed the phenological diversity among wine cultivars in response to a consistent difference of 1.5°C, indicated that the genetic diversity of grapevines is the key to adapting viticulture to warmer climates/periods (Gashu et al., 2020).

As fruits transpire only sparingly, their ability to regulate surface temperature is limited, thus they commonly experience sunburn, dehydration, photo-oxidative damage, berry shriveling, and metabolite disorders when exposed to elevated air temperatures or excessive solar irradiance (Greer and Weston, 2010; Krasnow et al., 2010; Reshef et al., 2019; Rustioni et al., 2020). Upon such environmental stress, fruits use complex mechanisms to maintain their development and protect themselves from damaging processes. These mechanisms include osmotic adjustments and the accumulation of UV protectants and the free radical scavengers, ascorbate (Asc), glutathione (GSH), pyridine nucleotides, carotenoids, and phenylpropanoids (Weisshaar and Jenkinst, 1998; Baumes et al., 2002; Winkel-Shirley, 2002; Kamffer et al., 2010; Foyer and Noctor, 2011; Noctor et al., 2012; Decros et al., 2019; Karniel et al., 2020). Nevertheless, the balance between oxidant and antioxidant chemical production in fruit can be disrupted in a harsh environment, leading to cellular damage due to the overproduction of reactive oxygen species (ROS; Gill and Tuteja, 2010; Decros et al., 2019), with a consequent negative effect on fruit metabolism and commercial quality.

In wine grapes, phenylpropanoids and carotenoids (precursors of C_13_ norisoprenoids), in addition to their antioxidant properties, are of particular interest as precursors of aroma, astringency, bitterness, and other mouth-feel properties in wine (Baumes et al., 2002; Downey et al., 2006; Cohen et al., 2008; Teixeira et al., 2013). Their levels in developing fruit are regulated by environmental conditions, developmental stage, and cultivar characteristics (Oliveira et al., 2004; Young et al., 2015; Joubert et al., 2016; Reshef et al., 2018).

Air temperature affects the metabolism of grapevine fruit (Cohen et al., 2008; Del-Castillo-Alonso et al., 2016; Gouot et al., 2019b; Yan et al., 2020). The impact of temperature on phenylpropanoids has already been studied in several wine grape cultivars (Cohen et al., 2008; Pastore et al., 2017; Gaiotti et al., 2018). For example, high temperatures have been found to reduce the concentrations of flavonols and anthocyanins (Pastore et al., 2017) and increase carotenoid content (Chen et al., 2017). Radiation and temperature have also been reported to affect the diurnal change of berry primary and secondary metabolites (Reshef et al., 2017, 2018, 2019); radiation was shown to trigger phenylpropanoid biosynthesis, whereas high temperature was shown to accelerate their degradation (Tarara et al., 2008; Azuma et al., 2012). Similarly, bunch exposure to direct sunlight has been shown to inhibit carotenoid gene expression and even accelerate carotenoid degradation (He et al., 2020). In contrast, elevated temperature has been shown to increase berry carotenoid concentration (Marais et al., 1991; Chen et al., 2017). These lines of evidence suggest that a substantial knowledge gap exists regarding carotenoid metabolism in response to air temperature in grapevine.

The primary objective of the present study was to examine the modulating effect of environment × cultivar interactions on berry secondary metabolism, carotenoid metabolism, and berry redox status (H_2_O_2_), under field conditions in warm, arid regions. In a recent study on grapevine (Gashu et al., 2020), we observed that white cultivars had an earlier and shorter ripening phase, partially avoiding the summer heat, in contrast to red cultivars in the same region. These results raised the possible importance of the duration of the ripening phase in determining fruit quality. In the current study, we chose the same 10 white cultivars to address the following question: does the air temperature/radiation regime during fruit ripening significantly alter berry phenylpropanoids and carotenoids? Accordingly, we used spectrophotometry and mass spectrometry (MS) to examine (i) the relationships of berry phenylpropanoids and carotenoids with climate indices, and (ii) the association between berry H_2_O_2_ and phenylpropanoids. We discuss the developmental changes in grape metabolic profiles and relate them to climate indices through network analyses.

## Materials and Methods

### Experimental Layout

The experiments were conducted over three consecutive seasons, from 2017 to 2019, in two vineyards 53 km apart in the Negev Highlands in Israel: the Mitzpe Ramon (MR) vineyard (30°38′48.6″N 34°47′24.5″E, 850 m asl) and the Ramat Negev (RN) vineyard at the Desert Agro-Research Center (30°58′43.4″N 34°42′31.6″E, 300 m asl). Average annual precipitation is 105 mm and 80 mm at MR and RN, respectively, occurring only in the winter (typically November through April), with considerable inter-annual fluctuations. Both vineyards shared the same experimental setup, comprising 10 white wine cultivars (Chardonnay, Chenin Blanc, Colombard, Gewurztraminer, Muscat Blanc, Muscat of Alexandria, Pinot Gris, Sauvignon Blanc, Riesling, and Semillon), grafted onto 140 RU rootstock; both vineyards were planted in 2012 in a randomized block design with four replicates of 8–9 vines each. The soils at both sites are sandy loam.

In all cultivars, yield was directed to about 5 kg vine^–1^. This was achieved through winter pruning to about 32 fruit-buds per vine, followed by thinning of excess fruiting shoots toward bloom, and further thinning of excess bunches when the actual clusters’ number and size were clear. Final yield adjustments took place where necessary, soon after véraison. Subsequently, the fruit yield usually ranged from 4.2 to 5.8 kg vine^–1^, with very few exclusions, and with minimum influences on fruit quality. Employing the vertical shoot positioning, canopy size was leveled throughout the vineyard using the following methods: (i) at the vegetative phase, shoots’ length was consistently confined by topping to about 2.2 m above ground; (ii) most of the blind shoots were removed; and (iii) when berries reached pea-size among most of the cultivars, deficit irrigation was practiced to avoid further shoot growth and branching. The experimental layout and meteorological data measurement have been briefly described elsewhere (Gashu et al., 2020).

Daily degree days (DDD) were calculated following Jones et al. (2010); the sum of DDD from véraison to harvest was computed for each cultivar to assess the DDD effect on fruit quality parameters, as follows:


(1) $$\text{DDD}=\sum_{\text{H}\,a\,r\,v\,e\,s\,t}^{\text{V}\,e\,r\,a\,i\,s\,o\,n}\text{max}\,\left[\left(\frac{\left[\text{T}\,m\,a\,x+T\,m\,i\,n\right]}{2}\right)-10,0\right]$$

where *Tmax* is the daily maximum air temperature and *Tmin* is the daily minimum air temperature.

To evaluate the effect of maximum and minimum air temperatures on fruit metabolism, we introduced accumulated degree hours indices expressing heat stress (Hs) and relaxation (Relx). Hs and Relx were calculated for each cultivar from véraison to harvest at which the hourly air temperatures were higher than 30°C and lower than 20°C, respectively.


(2) $$H\,s=\Sigma_{H\,a\,r\,v\,e\,s\,t}^{V\,e\,r\,a\,i\,s\,o\,n}\,m\,a\,x\,\left[\left(T\,e\,m\,p-30\right),0\right]$$

(3) $$R\,e\,l\,x=\Sigma_{H\,a\,r\,v\,e\,s\,t}^{V\,e\,r\,a\,i\,s\,o\,n}\,m\,a\,x\,\left[\left(20-T\,e\,m\,p\right),0\right]$$

where *Temp* is hourly air temperature.

### Berry Sampling and Metabolite Extraction

During each season, berries were sampled for metabolite extraction and berry indices at véraison and harvest. At véraison, each cultivar was sampled when berries reached approximately 50% softening (estimated weekly in eight tagged representative clusters per replicate). Only softened berries were sampled. The range of sampling dates varied from 1 to 7 days at MR and 1 to 14 days at RN. Toward harvest, berries were sampled from each cultivar approaching the 20 ± 1 °Brix level, with sampling date varied from 7 to 14 days at MR and up to 21 days at RN depending on the season. To minimize the effect of circadian rhythm, berries were sampled at 09:00. Samples were collected from four biological replicates at each location from each cultivar. In each sampling, at least 30 berries per replicate were pooled from five different vines in each block on the east side of the vine (six berries per vine were sampled from the top, middle, and bottom of the bunch), and immediately snap-frozen in liquid nitrogen. Berries were peeled while still frozen, by carefully separating the skin from the pulp, and the seeds were removed. The skin was kept at −80°C until further analysis.

Grape skin samples were lyophilized and ground under liquid nitrogen using a Retsch-mill (Retsch, Haan, Germany) with pre-chilled holders and grinding beads. For metabolite extraction, 40 mg of frozen skin powder were weighed and extracted in a 1-mL pre-cooled methanol:chloroform:water extraction solution (2.5:1:1 v/v) with ampicillin (1 mg mL^–1^ in water) and corticosterone (1 mg mL^–1^ in methanol) as internal standards (Hochberg et al., 2013; Degu et al., 2014). Skin extracts were filtered (0.22 μm Millipore, Burlington, MA, United States) and transferred to glass vials for analysis using ultra-performance liquid chromatography coupled to a quadrupole time-of-flight mass spectrometer (UPLC QTOF-MS; Waters, Burlington, MA, United States) operating in negative and positive ion modes.

### Liquid Chromatography-Mass Spectrometry Conditions

Chromatographic separation was performed using an Acquity UPLC BEH C_18_ column (100 mm × 2.1 mm, 1.7 μm) (Waters MS Technology, Manchester, United Kingdom) maintained at 40°C. The autosampler was maintained at 10°C. Leucine encephalin, at a concentration of 0.4 ng L^–1^, was used for lock mass calibration, in 50/50 acetonitrile/H_2_O with 0.1% v/v formic acid. The mobile phase was adjusted from 95% water, 5% acetonitrile, 0.1% formic acid (phase A) to 0.1% formic acid in acetonitrile (phase B), with the gradient transitioning from 100 to 60% phase A (0–8 min), 60–0% phase A (1 min), a gradual return to 100% phase A (3.5 min), and conditioning at 100–60% phase A (2.5 min), with a total run time of 15 min. The MS conditions were exactly as described previously by Hochberg et al. (2013). Briefly, the MS conditions were set as follows: Capillary voltage +3.0 keV; sampling cone voltage 27 V; extraction cone voltage 4 V; source temperature: 120°C; desolvation temperature: 300°C; cone gas flow: 50 L h^–1^; desolvation gas flow: 650 L h^–1^; collision energy: 6 eV, and for MS/MS spectra, collision energies were set from 25 to 50 eV; the scan range was set at 50–1,500 m/z; and the dynamic range enhancement mode was off.

### Liquid Chromatography-Mass Spectrometry Data Processing and Annotation

MassLynx™ version 4.1 (Waters) was used for system control and data acquisition. Metabolites were annotated based on fragmentation patterns searched against those in the ChemSpider metabolite database^1^, and the consistency of their retention times with those of identified metabolites was compared with the data in the scientific literature. A targeted metabolite profiling approach was used, and 36 metabolites were annotated uniquely in both negative (29 metabolites) and positive (seven metabolites) ion modes. The metabolite data generated by liquid chromatography-mass spectrometry (LC-MS) comprised unique mass intensity (mass-to-charge ratio) values (height) for each annotated compound. To minimize differences due to separate injection periods, we ran a bulked extraction of all samples (pool) four times in each batch as a reference throughout the injection sets, to enclose all metabolic variability of the different cultivars. The raw data for each metabolite in each batch were then normalized by dividing each value by the mean of the pool in the corresponding data file generated from each chromatogram.

### Spectrophotometric Assays for Hydrogen Peroxide, Glutathione, Ascorbic Acid (Ascorbate), Total Carotenoid, and Chlorophyll Measurements

A modified method of Noctor et al. (2016), was employed to measure H_2_O_2_ content in the berry. Eight to ten berries were sectioned while still frozen, and seeds were removed. The skin and pulp fractions were manually crushed with a mortar and pestle under liquid nitrogen. For H_2_O_2_ extraction, 100 mg of frozen powder were weighed and extracted in 2-mL of 1 M perchloric acid (HClO_4_) containing polyvinylpyrrolidone (5%). The samples were then centrifuged for 10 min at 14,000 RPM (microcentrifuge 5417R) at 4°C. The supernatant was neutralized with 5 M potassium carbonate (K_2_CO_3_) in the presence of 50 μL of 0.3 mM P-buffer (composed of monobasic dihydrogen phosphate and dibasic monohydrogen phosphate) and centrifuged for 1 min at 14,000 RPM, and the supernatant was decanted into new tubes. The supernatants were mixed in a reaction mixture comprising of 8.5 mM 4-aminoantipyrine (APP), 3.4 mM sodium 3,5-dichloro-2-hydroxybenzenesulfonate (BHS), 45 U mL^–1^ horseradish peroxidase (HRP) in 2 mL of 50 mM tris buffer (pH 7.5). Briefly, 40-μl samples of the supernatant were mixed with tris:APP:BHS:HRP (5:1:1:1 v/v) on a microplate (Epoch BioTek Instruments Inc., Burlington, MA, United States, and later Tecan Infinite^®^ M200, Tecan Austria GmbH, Salzburg, Austria). After 30 min of mixing the supernatant samples with the reaction mixture the H_2_O_2_ was measured spectrophotometrically at 510 nm, and then quantified using a standard calibration curve.

For total carotenoid and chlorophyll extraction, 20 mg of skin powder were weighed and extracted in 0.5 mL of 99% ethanol and stored in a dark room for 48 h at 4°C. Carotenoids and chlorophyll contents were determined spectrophotometrically at 470, 649, and 665 nm, respectively, in 200-μl samples of supernatant. The values obtained at the three wavelengths were used to calculate total carotenoid and chlorophyll content as described by Wintermans and De Mots (1965).

Since H_2_O_2_ measurements were consistent across the seasons, the 2018 samples were used for evaluating the other major redox buffers such as glutathione and Asc to explore the redox adjustment in white grapevine fruits. The samples were analyzed in bulks of the four replicates × cultivar × location. The method of Tietze (1969) was employed with minor modification to measure glutathione content in the berry skin tissue. For sample processing, 15 mg of lyophilized frozen skin powder were weighed and extracted in 1 mL of 1 M HClO_4_, and the reduced (GSH) and oxidized (GSSG) glutathione were measured as described by Oshanova et al. (2021). The Asc level in the berry skin tissue was measured as described by Jagota and Dani (1982). Briefly, 10 mg of lyophilized frozen skin powder were weighed and extracted in 800 μl of 10% trichloroacetic acid. The samples were then vortexed and centrifuged for 20 min at 14,000 RPM (microcentrifuge 5417R) at 4°C, and the supernatant was decanted into new tubes. The supernatants (400 μl) were mixed in a reaction mixture comprised of 1.6 mL DDw, and 200 μl of 10 fold diluted folin reagent. After 10 min of mixing the supernatant samples with the reaction mixture, Asc was measured spectrophotometrically at 760 nm, and then quantified using a standard calibration curve.

### Statistical Analyses

All statistical analyses were performed on log-transformed data using “R” version 3.6.0 (R Development Core Team, 2017). A three-way factorial analysis was used to assess the effects of cultivar (C), location (L), and growing season (Y), and the interactions between them, using the built-in *aov* function. The differences between locations for each cultivar were tested using the *Wilcox.test* function. Histograms were created using the *hist* function in the “ggplot2” package. Clustered heatmaps were created using *ComplexHeatmap* (Gu et al., 2016). Clustering of samples was calculated by Euclidean distances and the Ward.D2 clustering method in the functions *get_dist* and *hclust*, and the built-in “dendextend” and “factoextra” packages (Galili, 2015). Correlation-based network analyses were performed using the MetScape application and the NetworkAnalyzer tool, available in Cytoscape version 3.7.2. All correlation analyses and preparation for network visualizations were generated in “R” using the built-in cor function with the “Pearson” algorithm. Correlations were incorporated into the network if they were statistically significant (*p* < 0.05) and their correlation coefficient (r) was higher than 0.3 or lower than −0.3. Principal component analyses (PCA) were plotted using MetaboAnalyst version 4.0 (Chong et al., 2018) and JMP^®^ version 13 (SAS Institute Inc., Cary, NC, United States, 1989–2007). The norm of reactions that represents phenotypic changes (*y*-axis) due to environmental changes (*x*-axis), was calculated using linear regression models for each trait computing the slope. The slope represents phenotypic plasticity.

## Results

### Climatic Conditions in the Vineyards

The two experimental vineyards differed in their meteorological conditions (Gashu et al., 2020). On average, the RN vineyard experienced higher average, maximum and minimum air temperatures, despite the slightly lower incoming solar radiation. In addition, consistent 1.3-MJ m^–2^ day^–1^ difference in incoming solar radiation and 1.5°C difference in the daily mean temperature between the locations were maintained during all three seasons. Meteorological conditions between sites have been briefly described elsewhere (Gashu et al., 2020).

### The Differences Between Locations in Total Carotenoid Content Were Predominantly Affected by the Climate × Season Interaction

Cultivar (C), location (L), and seasonal (Y) factors contributed significantly to the measured differences in carotenoid content, while the non-significant contributions of location at harvest, and the C × L interaction at véraison were exceptions (Supplementary Table 1).

**FIGURE 1:**
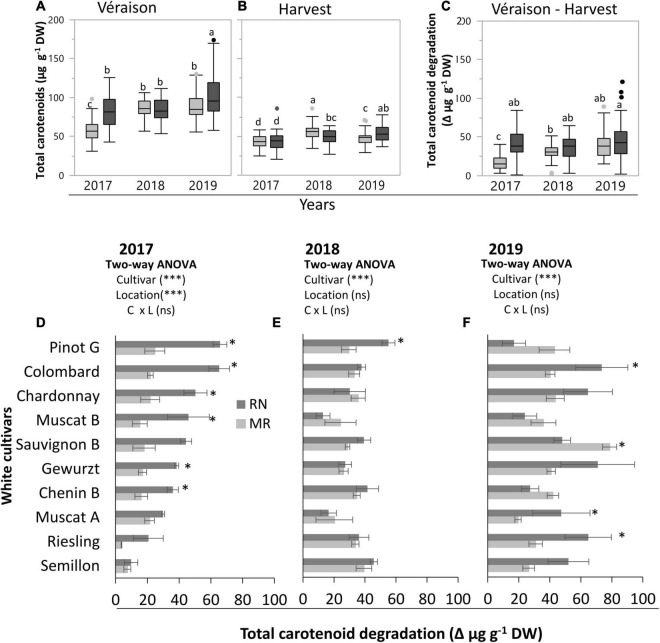
Total carotenoid content at véraison **(A)** and harvest **(B)**, and its degradation **(C–F)** (measured as the difference between véraison and harvest) in white skin berries at the Mitzpe Ramon (MR) and Ramat Negev (RN) vineyards during 2017–2019. Box plots show the difference in the mean of all cultivars (*n* = 4 replicates × 10 cultivars) between locations in each season (see Supplementary Table 1). Gray and black colors indicate cultivars grown at Mitzpe Ramon and Ramat Negev, respectively. Different lowercase letters indicate significant differences in cultivar means between locations. The bar graphs **(D–F)** show the effect of location and cultivar on carotenoid degradation in the 2017, 2018, and 2019 seasons. Error bars are standard error (*n* = 4). Bar plots followed by * indicate significant differences between locations (*P* < 0.05) within the same cultivar based on a non-parametric *t*-test. ^***^ indicates significant effects (*P* < 0.0001) of the main factors in each season based on a two-way ANOVA. C, cultivar; L, location; ns, not significant.

Testing each season independently revealed significant effects of cultivar and location in each season, except for ripe berries in 2017 and véraison berries in 2018, which were not affected by location (Supplementary Table 1). At véraison, the mean total carotenoid content ranged from 58 to 102 mg g^–1^ DW, with considerable variation among cultivars, locations, and seasons (Figure 1A and Supplementary Table 1). It was generally significantly higher at RN than MR in both 2017 and 2019 seasons, whereas in 2018, location had no effect (Figure 1A and Supplementary Table 1). Comparing seasons, the highest average total carotenoid content (92.8 mg g^–1^ DW) was measured in 2019. Among cultivars, Gewürztraminer (in 2019) displayed the highest carotenoid level (130.8 mg g^–1^ DW) at the warmer RN site across all seasons. In contrast, the lowest (43.4 mg g^–1^ DW) carotenoid level was measured in Semillon berries at MR in 2017 (Supplementary Table 1).

At harvest, the average carotenoid content across all cultivars ranged from 43.1 to 56.2 mg g^–1^ DW, with 2018 and 2019 seasons showing the greatest differences between locations (Figure 1B and Supplementary Table 1). In 2017, the carotenoid content was predominantly affected by cultivars, while in other years, it was significantly affected by location, being higher at MR than RN in 2018, but higher at RN than MR in 2019. Both the lowest (27.2 mg g^–1^ DW in 2017) and the highest (75.1 mg g^–1^ DW in 2019) carotenoid levels were measured at the warmer RN site for the Colombard and Pinot Gris cultivars, respectively (Supplementary Table 1).

### The Level of Carotenoid Degradation Is Dependent on the Topo-Climatic Differences Between Locations During Fruit Ripening

The average total carotenoid degradation, measured as the difference between véraison and harvest across all cultivars, varied significantly between locations, being consistently higher at RN (Figure 1C). The difference between locations was greater in the 2017 season. In the 2018 and 2019 seasons, the difference between locations was not significant (Figure 1C). Comparing seasons at MR, the mean total carotenoid degradation across all cultivars was 1.8 fold and 2.4-fold greater, in 2018 and 2019, respectively, than in 2017. In contrast, at RN, the inter-seasonal variation in carotenoid degradation was not significant.

In addition, degradation was also cultivar-dependent, with significant variation measured between locations for many cultivars in the 2017 and 2019 seasons (Figures 1D,F). The highest values were displayed at RN, in 2017, by Chenin Blanc, Gewürztraminer, Chardonnay, Colombard, and Pinot Gris (36.0–65.7 mg g^–1^ DW) (Figure 1D). Interestingly, only Pinot Gris showed a similar trend in 2018 (Figure 1E). In 2019, Muscat of Alexandria, Riesling, Gewürztraminer, and Colombard exhibited significantly higher degradation (47.4–73.5 mg g^–1^ DW) at RN than at MR. Sauvignon Blanc at RN was an exception among the cultivars, showing the lowest degradation in 2019 (Figure 1F).

**FIGURE 2:**
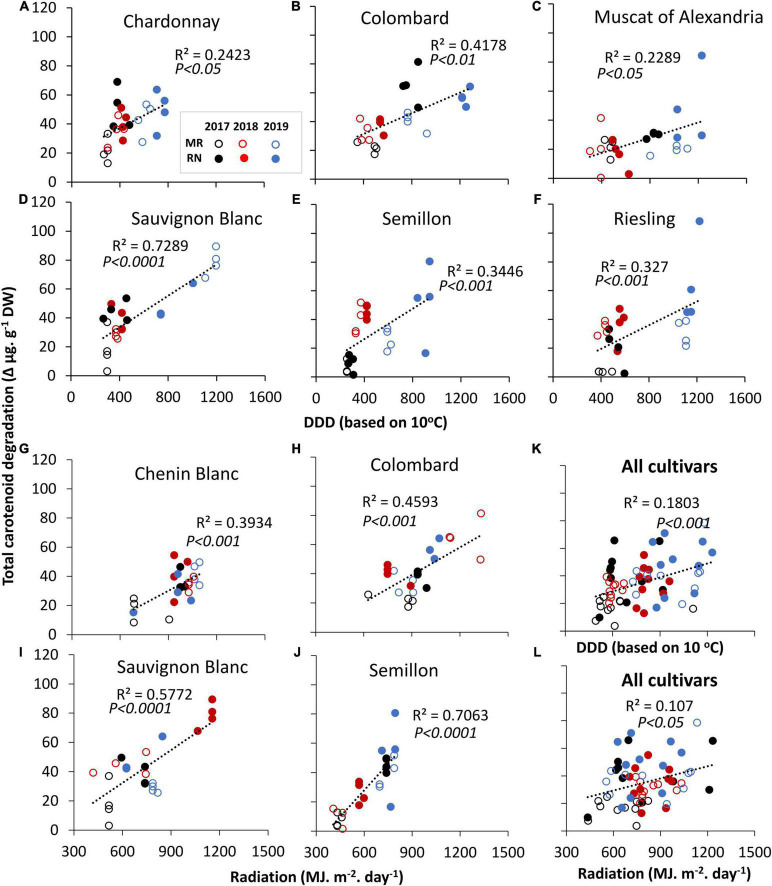
Linear correlation of total carotenoid degradation against accumulated daily degree days (DDD) **(A–F)** and daily summed radiation **(G–J)** that were calculated from véraison to harvest in white cultivars grown at Mitzpe Ramon (MR) and Ramat Negev (RN) during 2017–2019. **(K,L)** Represent correlations of total carotenoid degradation across all cultivars (*n* = 4 replicates × 10 cultivars × 2 locations × 3 seasons) with DDD and radiation, respectively. In each season, data are values of four biological replicates. Only significant correlations are shown. Circles denote the MR (open) and RN (closed) vineyards. Colors indicate cultivars grown in 2017 (black), 2018 (red), and 2019 (blue). See Supplementary Table 2.

The sums of daily degree days (DDD) and radiation from véraison to harvest were correlated against total carotenoid degradation (Figures 2A–J). The results showed that an increase in either DDD or radiation was associated with greater carotenoid degradation (Figures 2K,L), and the relationship was cultivar-dependent. A significant correlation with DDD was found in Chardonnay, Colombard, Muscat of Alexandria, Sauvignon Blanc, Semillon, and Riesling (Figures 2A–F). Sauvignon Blanc displayed the strongest correlation (*p* < 0.0001, *R*^2^ = 0.73) (Figure 2D). Similarly, an increase in the cumulative radiative flux was significantly associated with carotenoid degradation (*p* < 0.05), but the relationship was generally weaker (Figure 2L). Nevertheless, degradation in Sauvignon Blanc and Semillon exhibited a significant correlation with radiation (*R*^2^ = 0.56 and 0.71, respectively, *p* < 0.0001) (Figures 2I,J). Colombard, Sauvignon Blanc, and Semillon were affected by both DDD and radiation. In Gewürztraminer, Muscat Blanc and Pinot Gris, carotenoid degradation was not significantly correlated with either factor (Supplementary Table 2).

### Cultivar-Specific Response of Phenylpropanoid Metabolism to Climate Differences

**FIGURE 3:**
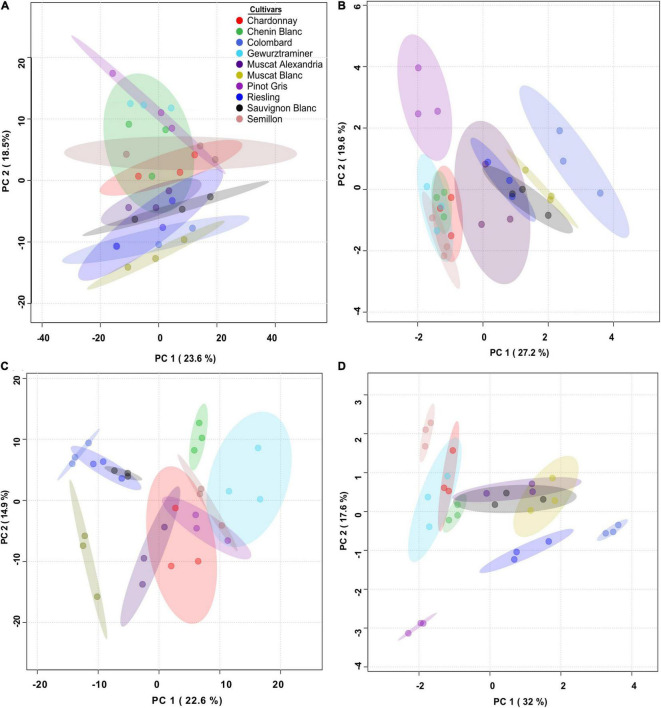
Overview of the differences between cultivars in skin phenylpropanoids at véraison (left) and harvest (right) grown at the Mitzpe Ramon **(A,B)** and Ramat Negev **(C,D)** vineyards during 2017–2019. Principal component analyses (PCAs) were plotted using mean values of four biological replicates (*n* = 4) in each season. PCAs for véraison samples were generated using pareto-scaled data. PCAs for harvest samples were plotted using log-transformed and pareto-scaled data. Samples are colored by cultivar names.

Thirty-four secondary metabolites and four amino acids were identified in the skin of 10 different white wine grapevine cultivars at véraison and harvest. To explore the overall variability of skin phenylpropanoid accumulation in each location, UPLC- QTOF MS-generated metabolite profiles were plotted in a principal component analysis (PCA). The analysis revealed a cultivar-specific response to location, particularly at véraison. At RN, the separation between varieties was better resolved on PC1 and PC2 (Figures 3A–D). For example, Colombard, Riesling, and Sauvignon Blanc at véraison clustered together and were separated from other cultivars due to relatively lower levels of taxifolin (dihydroquercetin), astilbin, and naringenin chalcone-4*-O-*glucoside (naringenin ch-4-glu) at this time point (Figure 3C and Supplementary Figure 1). However, these cultivars were well separated from each other later at harvest (Figure 3D).

In contrast, the MR vineyard was characterized by greater variability at harvest (Figures 3A,B). At this time point, the PCA of the metabolic data showed that cultivars separated along PC1 and PC2 due to the contribution of astilbin, taxifolin, and naringenin-4-glu, myricetin and its aglycone, and stilbenes (Figure 3B and Supplementary Figure 1). Taxifolin, astilbin, and naringenin ch-4-glu accumulated to relatively higher levels mainly in Chardonnay, Chenin Blanc, Gewürztraminer, and Semillon (Supplementary Figures 1A,B), regardless of location.

The analysis also emphasized commonalities between the two experimental vineyards. At harvest, for example, in both locations, Colombard and Pinot Gris were grouped away from the other cultivars (Figures 3B,D), mainly because of stilbenes in the former and of myricetin and its conjugated forms in the latter (Supplementary Figure 1B).

### Cultivar-Specific Differences in Skin Phenylpropanoids Reflect the Cultivar × Environment Interaction in Response to the Climate Differences Between Locations

**FIGURE 4:**
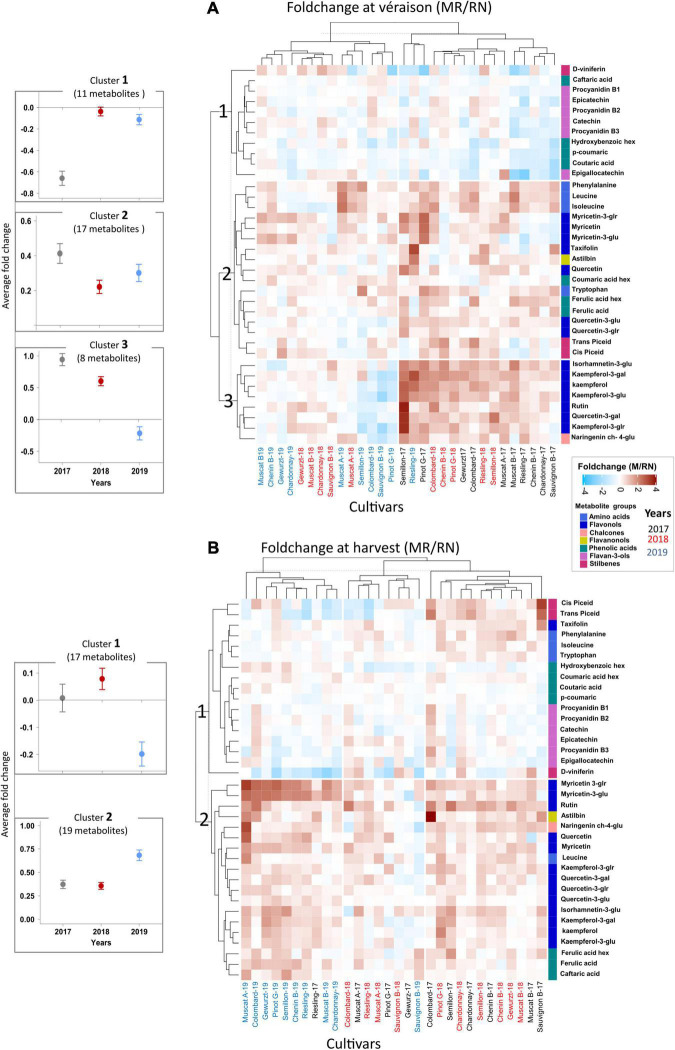
The variability in skin phenylpropanoids at véraison **(A)** and harvest **(B)** between locations during 2017–2019, expressed as the foldchange of average response values [Mitzpe Ramon (MR)/Ramat Negev (RN)]. The mean value of each metabolite (*n* = 4 biological replicates) for each cultivar in each location and season was calculated separately. Then, the MR values were divided by the RN values and transformed by log2. The heatmap was generated using the log2-transformed data. Euclidean distance was used for hierarchical clustering dendrogram. Cultivar names are denoted by vintage abbreviations (17, 18, 19). Colored cultivar names indicate samples collected in 2017 (black), 2018 (red), and 2019 (blue). Colored rectangles represent metabolite increases at MR (red) and RN (blue). A mirror heatmap of significance values is presented in Supplementary Figure 2. The dot plots (left) indicate the average foldchange (±SE) of metabolites in each representative cluster in the 2017, 2018, and 2019 seasons.

Cultivar, location, season, and their interaction all had a significant impact on berry phenology and primary metabolism (Gashu et al., 2020). In a similar manner, skin phenylpropanoids at both véraison and harvest were significantly affected by cultivar, location, season, and their interactions (Supplementary Tables 3, 4). A hierarchical clustering (HCL)-based heatmap visualization of the fold changes (MR/RN) in metabolite level (Figure 4) shows the cultivar-related differences between locations; however, there was a seasonal element, as indicated by the significant L × Y interaction (Supplementary Tables 3, 4).

At véraison, the HCL heatmap analysis on the fold change data produced three main metabolite clusters depending on the degree of differences between locations (Figure 4A). The first cluster (cluster 1) included metabolites that markedly accumulated at RN, e.g., flavan-3-ols, the phenolic acids, caftaric acid, hydroxybenzoic hexoside, p-coumaric and coutaric acid, and the stilbene, D-viniferin. Clusters 2 and 3 included mainly flavonols, flavanonols, naringenin ch-4-glu, other stilbenes and amino acids, all highly accumulated at MR. Exceptions among flavonols (cluster 3) were the lower levels of kaempferol and its aglycones, rutin, and quercetin-3-galactoside in Semillon, Colombard, Sauvignon Blanc, and Pinot Gris at MR (2019) (Figure 4A and Supplementary Figure 2A). The relative abundance of naringenin ch-4-glu in these cultivars was also lower at MR than at RN in 2019.

At harvest, differences between locations were also evident, particularly in flavonols (Figure 4B). Cluster 1 included flavan-3-ols, amino acids, some phenolic acids, and stilbenes, which exhibited only minor differences between locations with very few exceptions among cultivars, e.g., a higher content of stilbenes in Sauvignon Blanc at MR in 2017, while Riesling had significantly higher stilbenes at the warmer RN site in the 2018 and 2019 seasons. Cluster 2 included metabolites with a remarkably higher content at MR, mainly flavonols and flavanonols. Results indicate that a strong C × Y interaction affected the metabolite profiles in each location (Figure 4B and Supplementary Table 4). For example, the astilbin foldchange between locations in Colombard (in 2017) was 4-fold greater than in Chardonnay, Gewürztraminer, and Pinot Gris in this year (Figure 4B and Supplementary Figure 2B). In contrast, only a minor location effect was observed for the phenylpropanoid metabolism of Sauvignon Blanc (in 2019 and 2018) and Gewürztraminer (in 2017).

The change in skin phenylpropanoids from véraison to harvest considerably differed between locations; however, it was season-dependent (Supplementary Figure 3). Generally, higher degradation was measured at the warmer RN site, e.g., flavan-3-ols, rutin, quercetin-3-glucuronide and kaempferol-3- glucuronide among flavonols, and caftaric acid, p-coumaric, coutaric acid, and coumaric acid hexoside; nonetheless, the seasonal effect was considerable. The higher pace of reduction from véraison to harvest in leucine in Colombard, Sauvignon Blanc, and Muscat of Alexandria at both locations was an exception among amino acids (Supplementary Figure 3), which may possibly affect their aromatic components.

**FIGURE 5:**
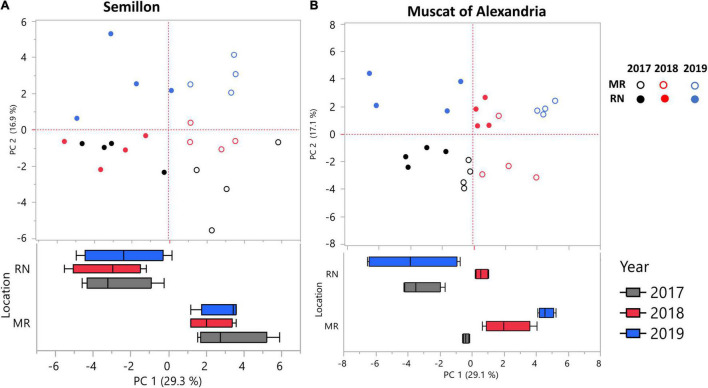
Principal component analysis (PCA) of skin phenylpropanoids at harvest for cultivars affected by location (L) and season (Y) **(A)**, and their interaction **(B)**. **(A)** PCA performed on skin phenylpropanoids of the Semillon cultivar shows that samples are significantly separated by their location, i.e., Ramat Negev (RN) or Mitzpe Ramon (MR). **(B)** PCA performed on skin phenylpropanoids of the Muscat of Alexandria cultivar shows that sample separations are significantly influenced by the L × Y interaction. Data were scaled to the median of each metabolite, and the value of four biological replicates (*n* = 4) in each season and location were used to plot the PCs. The PCA was first plotted for each cultivar, and a two-way ANOVA model was performed using PCA scores for each cultivar separately. The analysis resulted in the identification of two subsets of cultivars; (i) cultivars affected by location and seasons, and (ii) cultivars affected by L × Y interactions. Only one representative cultivar, **(A,B)** from each group is depicted. Box plots were generated using the first PC scores.

In an effort to identify cultivars that are sensitive to topo-climatic variation, PCs were analyzed and plotted for each cultivar separately, using phenylpropanoid harvest data from 2017, 2018, and 2019. A two-way ANOVA was performed using PCA scores for each cultivar. The analysis resulted in two cultivar groups (Figures 5A,B, Supplementary Table 5): (i) location sensitive cultivars: Muscat Blanc, Riesling, Semillon and Sauvignon Blanc, and (ii) cultivars affected by the L × Y interaction: Chardonnay, Chenin Blanc, Gewürztraminer, Colombard, Muscat of Alexandria, and Pinot Gris.

To understand the cultivar versus environmental contribution to the observed metabolite changes, we calculated the norm of reaction for all traits (Supplementary Figure 4). In the norm of reaction plot, the slope represents phenotypic plasticity; thus, the higher the slope, the greater the phenotypic plasticity of a particular genotype for a particular trait in response to the environment. Among metabolites, myricetin-3-glucuronide exhibited the steepest slope (Supplementary Figures 4A,B), indicating that the differences between MR and RN sites were largely attributed to environmental variation.

### A 1.5°C Warmer Topo-Climate Imposes Higher Oxidative Stress on Grapevine Cultivars, but Is Differentiated by Genotype

**FIGURE 6:**
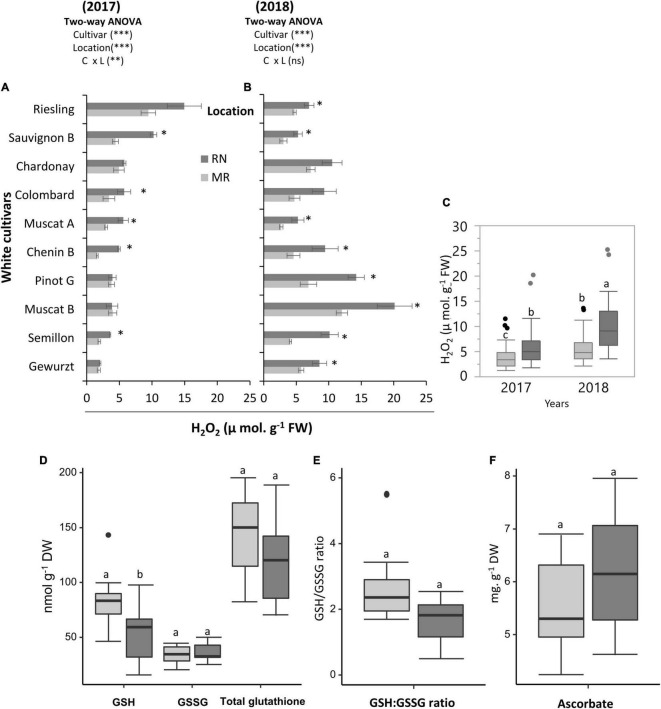
Cultivar and environmental effect on H_2_O_2_ and major redox buffers in ripe white berries. H_2_O_2_ content measured on ripe white berries grown at the Mitzpe Ramon (MR) and Ramat Negev (RN) vineyards in 2017 **(A)** and 2018 **(B)**. Error bars are standard error (*n* = 4). Bar plots followed by * indicate significant differences between locations (*P* < 0.05) within the same cultivar based on a non-parametric *t*-test. ^**^P < 0.01, ^***^P < 0.001 indicates Significant effects of the main factors in each season based on a two-way ANOVA. The box plot **(C)** shows the difference in the mean H_2_O_2_ content of all cultivars (*n* = 4 replicates × 10 cultivars) between locations in the 2017 and 2018 seasons. **(D)** The differences in the mean glutathione content and GSH to GSSG ratio **(E)** of all cultivars (*n* = bulked replicate × 10 cultivars) between locations in the 2018 season. **(F)** The differences in the mean ascorbic acid content of all cultivars (*n* = bulked replicate × 10 cultivars) between locations in the 2018 season. Different lowercase letters in box plots indicate significant differences in cultivar means between locations. Gray and black colors indicate cultivars grown at Mitzpe Ramon and Ramat Negev, respectively.

A statistical analysis of H_2_O_2_ in berries revealed significant effects of cultivar, location, season, and the C × L and C × Y interactions (Supplementary Table 4). In seasons 2017 and 2018, the cultivar mean of H_2_O_2_ was greater at RN than at MR (Figure 6C). Here, cultivars such as Sauvignon Blanc, Muscat of Alexandria, and Semillon, exhibited significant differences between locations in H_2_O_2_ content in both seasons (Figures 6A,B). In addition, the cultivar factor significantly affected (*P* < 0.05) H_2_O_2_, with accumulation greater in Riesling (14.9 μ mol. g^–1^ FW) and Sauvignon Blanc (10.2 μ mol. g^–1^ FW) berries in 2017, and Muscat Blanc (20.1 μ mol. g^–1^ FW) and Pinot Gris (14.2 μ mol. g^–1^ FW) in 2018 at RN. The lowest H_2_O_2_ content was measured at the MR vineyard for Chenin Blanc, Semillon, and Gewürztraminer (Figures 6A,B).

Since the H_2_O_2_ content was consistent across seasons, the 2018 samples were used for evaluation of glutathione and Asc in order to get a better picture of the redox adjustment in the fruits. The cultivar mean of GSH was greater at cooler MR than at warmer RN (Figure 6D). Cultivars such as Chenin Blanc, Colombard, and Semillon, exhibited higher GSH content at MR than at RN (Supplementary Figure 5). In addition, cultivars displayed great variability in GSH content at each site, with the lowest GSH content measured for Chenin Blanc (46.1 and 15.9 nmol g^–1^ DW in MR and RN, respectively) at both sites, whilst the highest content was measured for Semillon (143.3 nmol g^–1^ DW) at MR and Sauvignon (97.3 nmol g^–1^ DW) at RN (Supplementary Figure 5). The GSSG content in grape berry skin ranged from 20.1 to 50.3 nmol g^–1^ DW (Supplementary Figure 5) but the overall effect of location was not significant (Figure 6D). Relatively high GSH:GSSG ratio was observed at the cooler MR, a difference which was mainly attributed to higher GSH content at MR (Figure 6E). The GSH:GSSG ratio varied from 1.7 to 5.5 at MR, and from 0.5 to 2.5 at warmer RN (Supplementary Figure 5). In addition, 61% (at RN) to 71% (at MR) of the total glutathione in the skin tissue was in the reduced form. In spite of considerable variability between cultivars, Asc content was not affected by location (Figure 6F). The Asc content varied from 3.3 to 8.3 mg g DW^–1^, with the highest value measured for Chenin Blanc and lowest for Chardonnay and Semillon depending on location (Supplementary Figure 5).

### Flavonols Are Strongly Associated With the Oxidative Status (H_2_O_2_) of the Berries

**FIGURE 7:**
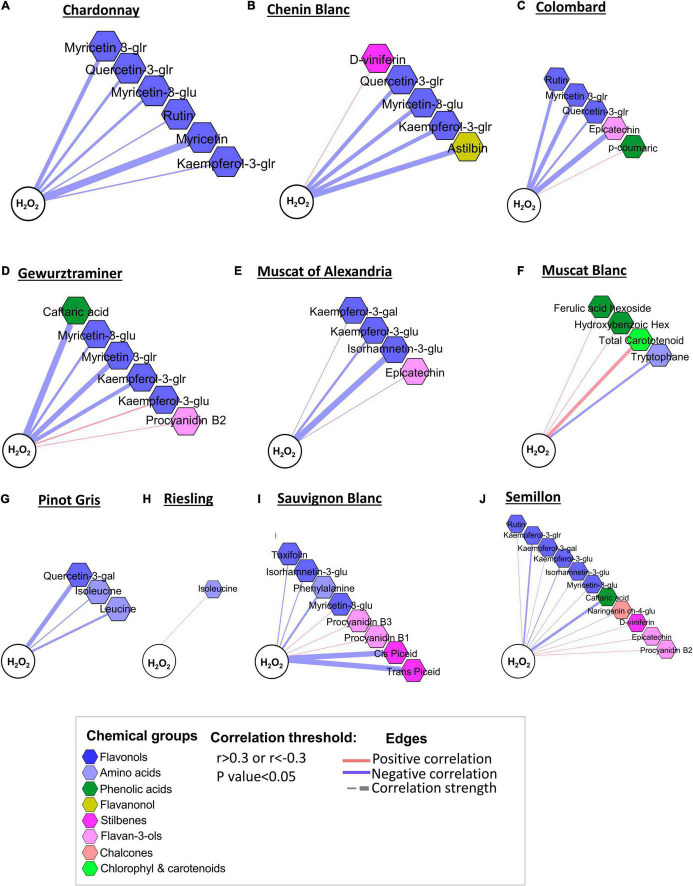
Network visualization of correlations between H_2_O_2_ and skin phenylpropanoids measured in 10 white grapevine cultivars **(A–J)** in 2017 and 2018. Metabolites are color-coded according to the chemical groups. The correlation analysis was performed using four biological replicates at the Mitzpe Ramon (MR) and Ramat Negev (RN) vineyards in each season; only significant correlations are presented. Positive correlations are shown as red edges, and negative correlations as blue edges. Correlations were based on Pearson’s method.

The oxidative status of the berries was correlated with the level of skin phenylpropanoids (*p*-value < 0.05 and *r* > 0.3 or *r* < −0.3), but this relationship was cultivar-specific (Figures 7A–J). An increase in the H_2_O_2_ level in Chardonnay, Chenin Blanc, Colombard, Gewürztraminer, Muscat of Alexandria, Sauvignon Blanc, and Semillon was associated with a lower content of flavonols, mainly glucuronide and glucoside (Figures 7A–D). The positive correlations of kaempferol-3-glucuronide and myricetin-3-glucoside with H_2_O_2_ in Gewürztraminer and Sauvignon Blanc, respectively, were exceptions among the flavonols (Figure 7D). Amino acids, such as tryptophan, leucine, and isoleucine, were negatively correlated with H_2_O_2_ in Muscat Blanc, Pinot Gris, Riesling, and Sauvignon Blanc, suggesting that at the warmer RN site, higher oxidative stress may lead to the degradation of important volatile precursors. In contrast, flavan-3-ols were generally positively correlated with H_2_O_2_. Among stilbenes, D-viniferin was positively associated with H_2_O_2_ in Chenin Blanc and Semillon (Figures 7B,J). In contrast, *cis*- and *trans*-piceid displayed strong negative correlations with H_2_O_2_ in Sauvignon Blanc (Figure 7I). Muscat of Alexandria, Muscat Blanc, Pinot Gris, and Riesling (Figures 7E–H) showed relatively loose metabolic relations with H_2_O_2_ compared to Sauvignon Blanc and Semillon (Figures 7I,J).

### Network Analysis Reveals the Differences Between Vineyards Within the Metabolite-to-Environmental Factor Correlation

**FIGURE 8:**
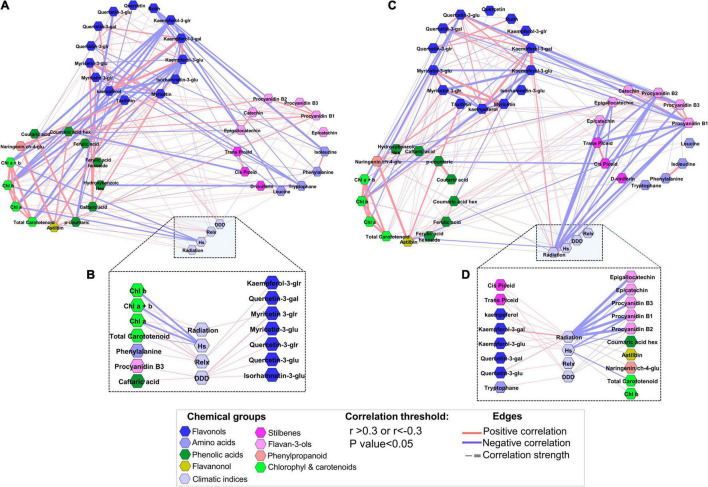
Network visualization of skin phenylpropanoid metabolites as analyzed from ripe white grapevine berries grown at the Mitzpe Ramon (MR) **(A)** and Ramat Negev (RN) **(C)** vineyards during 2017–2019. **(B,D)** Show metabolites significantly correlated with environmental factors (Hs, Relx, and radiation) at MR and RN, respectively. Metabolites are color-coded and clustered according to the chemical groups. Correlation analyses were performed using the mean value of four biological replicates of each cultivar in the 2017, 2018, and 2019 seasons at MR and RN separately; only significant correlations are presented. Positive correlations are shown as red edges, and negative correlations as blue edges. Correlations were based on Pearson’s method. Hs, heat stress degree hours; Relx, relaxation degree hours.

To further investigate the relationship of metabolites with temperature and radiation during ripening, a correlation-based network analysis was performed using the metabolite profiles at harvest for RN and MR samples separately. The results showed that the MR network was characterized by a slightly smaller number of edges (180) and a lower network density (0.19) than RN (194 and 0.21 edges and density, respectively) (Supplementary Table 6). With the threshold level set at *p*-value < 0.05 and *r* > 0.3 or *r* < −0.3, metabolite-to-metabolite and metabolite-to-environmental factor correlations were location-specific (Figure 8). Amino acids at MR were negatively correlated with flavonols (Figure 8A). Similarly, kaempferol and its aglycone were negatively correlated with carotenoid and chlorophyll pigments. It is noteworthy that only phenylalanine, among the amino acids, and procyanidin B3, among the flavan-3-ols, were correlated with climatic factors (Figure 8B). Flavonols were positively correlated with accumulated DDD. Chlorophyll and carotenoid pigments were positively correlated with accumulated relaxation degree hours (Relx) and negatively correlated with heat stress degree hours (Hs) (Figure 8B).

In contrast to the MR network, in the RN network, amino acids were not correlated with flavonols, but with astilbin and naringenin ch-4-glu (Figure 8C). A single positive correlation of tryptophan with quercetin-3-glr was an exception. The correlations of chlorophyll and carotenoid pigments with Relx were not significant (Figure 8D). Another significant difference between the two networks stemmed from flavan-3-ols, which were negatively correlated with kaempferol aglycones and quercetin-3-glu in the RN network, but not in the MR network. Of all metabolites correlated with environmental factors, flavan-3-ols showed a robust negative correlation with radiation (*r* < 0.5), while flavonols were positively correlated with radiation and accumulated heat load (Hs) (Figure 8D). Network analyses revealed that skin phenylpropanoids at the warmer RN site are tightly linked to the radiation and temperature regimes during fruit ripening, suggesting that the warmer region could experience a greater decline in berry quality, and potentially in wine quality, than the cooler region.

## Discussion

Carotenoids are considered to be important ROS quenchers (Ramel et al., 2012). Taking this into account and considering their importance in aroma development, an acute knowledge gap exists regarding carotenoid metabolism in response to temperature in grapevine. For instance, it was found that grapes with a high carotenoid content are produced in warmer regions (Marais et al., 1991; Chen et al., 2017). We show that differences between the warmer RN site and the cooler MR site were strongly dependent on genotype, developmental stage, and seasonal variations (Figure 1 and Supplementary Table 1). In line with what has been previously shown in this study, the mean carotenoid content of véraison berries was more pronounced at the warmer RN site in the 2017 and 2019 seasons. Likewise, the average carotenoid content of ripe berries was higher at RN than at MR in 2019; however, in 2018 it was higher at MR than at RN.

Carotenoids are synthesized from the first stage of fruit formation until véraison, and are degraded during ripening (Chen et al., 2017) to produce C_13_-norisoprenoid and other compounds (Baumes et al., 2002; Crupi et al., 2010). Young et al. (2015) found that a high carotenoid content at early stages was positively correlated with the volatile norisoprenoid level at later stages in light-exposed berries. However, exposing berries to direct sunlight was reported to reduce carotenoid content in the Bairrada and Vinho Verde region, Portugal (Oliveira et al., 2004). Our results indicate that: (i) overall, greater carotenoid degradation occurs at slightly higher temperatures (Figure 1), (ii) degradation is cultivar–specific, and (iii) it was positively correlated with the accumulated DDD and radiation from véraison to harvest, suggesting a synergistic effect of radiation and temperature on carotenoid degradation. Notably, the degradation of carotenoids in Gewürztraminer, Muscat Blanc, and Pinot Gris was not correlated with either factor, suggesting that the enzymatic apparatus responsible for carotenoid cleavage and regulatory genes may differ substantially among cultivars.

Plants, in general, produce ROS when exposed to environmental stresses (Carvalho et al., 2015; Xi et al., 2017). In grape berries, an oxidative burst occurs immediately after véraison due to the rapid accumulation of H_2_O_2_, resulting in a hastened fruit ripening process (Pilati et al., 2007, 2014; Xi et al., 2017). In our study, however, despite considerable differences between cultivars and seasons, the overall H_2_O_2_ content among cultivars tended to be higher at the warmer RN site and was associated with extended fruit ripening (Gashu et al., 2020), possibly a con-cause of oxidative stress severity. In addition, higher H_2_O_2_ content at the warmer RN site was accompanied by lower GSH content, which may explain why berries at the warmer site experienced high oxidative stress. In contrast, greater than 70% of the total glutathione in the grape berry skin tissue at the cooler MR was in the reduced form, which is an indication of better redox buffer (Foyer and Noctor, 2011). Our results are in contrast to Suklje et al. (2012), who found no increment of GSH content in Sauvignon Blanc grape juice in response to bunch exposure to direct sunlight. However, we believe that the climate component for each location played a role in the difference between the two studies. In other plant species, however, leaf glutathione increased in response to higher radiation, but was generally less responsive than ascorbate (Foyer and Noctor, 2011). Considering that there is no direct evidence showing that GSH can be synthesized in the berry (Kritzinger et al., 2013), lower GSH content at the warmer RN site could be attributed to a slower influx of GSH from source leaves to sink berries under elevated temperatures. A more detailed analysis of the interplay between leaf-photosynthesis and source-sink translocation under different temperature regimes is required to gain conclusive evidence on the regulatory mechanisms driving the redox machinery in grape berries.

In contrast to GSH, Asc content was not affected by location though its content was cultivar-dependent. In a recent study (Gashu et al., 2020), tartaric acid content, a product of Asc catabolism, was significantly higher in véraison berries at the warmer site. It is possible that high temperature could hasten Asc catabolism in the berries toward tartaric acid. Contrasting observations were reported in Shiraz grape berries, where the conversion of Asc to tartrate was shown not to be light-sensitive (Melino et al., 2011).

In different plant species, elevated temperature led to ROS overproduction, resulting in altered cellular metabolism and increased lipid peroxidation (Huan et al., 2016; Hasanuzzaman et al., 2016). Our results indicated that amino acids, flavonols, and stilbenes were inversely correlated with H_2_O_2_ content, suggesting that higher H_2_O_2_ content in the fruit could have led to degradation of important volatile precursors and might impair other important phenylpropanoid metabolites. This trend was characterized by considerable variability among cultivars. For instance, Pinot Gris and Riesling were more chemically resilient than Sauvignon Blanc and Semillon, indicating that cultivar diversity could be vital for high quality grape production in arid regions or in a future warmer climate scenario.

Elevated temperature is often reported to reduce anthocyanin accumulation in grapes (Cohen et al., 2008; Pastore et al., 2017; Arrizabalaga et al., 2018; Yan et al., 2020). In contrast, low night temperature (10–11°C) at véraison has been shown to enhance anthocyanin accumulation (Gaiotti et al., 2018). While much of the research on the effects of temperature has been dedicated to enhancing anthocyanin accumulation in red berries, its effect on white skin berry metabolites remains poorly understood. Our analyses show that phenylpropanoids in white skin berries were strongly influenced by cultivar, location, and season, where the warmer site was generally associated with lower phenylpropanoids. The analysis of the norm of reaction revealed (Supplementary Figure 4A) that the observed variance in metabolite content was trait-specific, i.e., for some metabolites such as myricetin and its aglycone the observed difference was due to the interaction between cultivar and environment, while for others, e.g., *trans*-piceid, the observed variance was specific to the variety.

In a previous study, prolonged pre-véraison and post-véraison intervals led to a higher risk of exposing clusters to recurrent heatwaves, which can lead to an imbalanced accumulation of precursors for aroma and other quality-related compounds in the fruit (Gashu et al., 2020). In support of this occurrence, we showed a diminished content of the amino acid phenylalanine (a precursor of phenylpropanoids), as well as tryptophan, leucine and isoleucine (precursors of aroma volatiles) in véraison berries at the warmer site. At harvest, results were similar, that is cultivar- and season-specific. These results are in contrast with other studies showing increased amino acid accumulation in the fruit in response to elevated temperature (Torres et al., 2017; Gouot et al., 2019a). Further research is needed to unravel the effect of temperature shifts on polyphenols and their precursors in the central metabolism, possibly by combining metabolite profiling and gene expression analysis.

Phenolic acids play important roles both in defense by protecting plants against biotic stress and as free radical scavengers when fruits are exposed to abiotic stresses (Proestos et al., 2006; Lafay and Gil-Izquierdo, 2008; Mandal et al., 2010; Teixeira et al., 2013). It has been reported that their synthesis, from the first stage of fruit formation until véraison, and their reduction in concentration, with an increase in ripeness (Teixeira et al., 2013), may conceal the influence of temperature on their content at harvest (Del-Castillo-Alonso et al., 2016). In line with what was shown by these studies, phenolic acids did not exhibit a strong correlation with climatic variables in the current study; however, significant differences between locations were found in both véraison and harvest berries, with the latter being more varietal-dependent.

Flavonols are ROS scavengers and UV-B screeners, and act as extreme temperature protectants when fruits are exposed to environmental constraints (Weisshaar and Jenkinst, 1998; Winkel-Shirley, 2002). The literature is inconsistent with respect to the impact of temperature on flavonol accumulation. For example, heat treatment from véraison to harvest reduced flavonol concentration in potted Sangiovese berries (Pastore et al., 2017), while in another study, high temperatures (28/18°C day/night) increased flavonol accumulation in Tempranillo berries (Torres et al., 2017). In the present study, flavonols and flavanonols generally decreased in RN berries both at véraison and harvest (Figure 4), suggesting a considerable metabolic response to arid environments, with increasing fruit susceptibility to oxidative stress in warmer regions.

Flavan-3-ols protect against biotic and abiotic stresses while contributing to fruit bitterness and astringency (Bogs et al., 2005; Ullah et al., 2017; Reshef et al., 2018). Despite their importance, little is known about environmental effects on their rate of production. Heat treatment after fruit set has been shown to significantly increase skin flavan-3-ol subunits, and subsequently, skin tannins, in Shiraz berries (Gouot et al., 2019a). In contrast, cold treatment from fruit set to véraison has been reported to increase epigallocatechin in Merlot berries (Cohen et al., 2012). This discrepancy can be explained by cultivar metabolic diversity. In the present study, significant variability between cultivars was measured; while RN-véraison berries had a generally higher flavan-3-ol content than MR-véraison berries, a greater reduction of these compounds was monitored from véraison to harvest at RN than at MR, resulting in smaller overall differences between locations at harvest. In addition, network analysis revealed that flavan-3-ols were inversely correlated with accumulated radiation and heat load during ripening (Figure 8B), likely contributing to the observed pattern of change in flavan-3-ols.

UV light treatment has been reported to trigger stilbene accumulation (Douillet-Breuil et al., 1999; Adrian et al., 2000). However, other studies have not found a significant difference in stilbene accumulation between sites (Dal Santo et al., 2016). In the present study, stilbenes (*cis*- and *trans*-piceid) were significantly lower in the RN berries, particularly at véraison, but were characterized by a cultivar × season interaction. Taken together, these lines of evidence emphasize the significant potential of cultivar plasticity to withstand elevated temperature and heat-induced oxidative processes.

## Conclusion

This detailed 3-year study on the oxidative status and carotenoid and phenylpropanoid contents of berries in 10 white grape cultivars provide evidence of the fundamental role of cultivar diversity in the production of high quality grapes in arid regions. Here, we showed that both radiation and temperature regimes during fruit ripening synergistically affect carotenoid and phenylpropanoid contents. However, the effect was seasonal and varietal dependent, and the differences in carotenoid and phenylpropanoid contents between sites were not always associated with differences in the length of fruit ripening phases. As a result, careful cultivar selection and strategies for controlled cluster microclimatic conditions must be considered in warm climates. Further research is required to unravel the biodiversity of the regulatory processes modulating carotenoid and phenylpropanoid degradation in *Vitis vinifera.*

## Data Availability Statement

The original contributions presented in the study are included in the article/Supplementary Material, further inquiries can be directed to the corresponding author.

## Author Contributions

AB and AF conceived and planned the study. KG, TA, CS, and AB collected the berry samples in the field. TA did carotenoid extraction. AD measured glutathione content. KG prepared the berry samples for extraction, performed the sample extraction and data analysis and analysis using the GC-MS device, and wrote the body of the manuscript with AF and AB. All authors reviewed and approved the manuscript.

## Conflict of Interest

AB was employed by Ramat Negev Works Ltd. The remaining authors declare that the research was conducted in the absence of any commercial or financial relationships that could be construed as a potential conflict of interest.

## Publisher’s Note

All claims expressed in this article are solely those of the authors and do not necessarily represent those of their affiliated organizations, or those of the publisher, the editors and the reviewers. Any product that may be evaluated in this article, or claim that may be made by its manufacturer, is not guaranteed or endorsed by the publisher.

## Footnotes

1
http://www.chemspider.com/

## Abbreviations

Myr-3-glr: Myricetin-3*-O*-glucuronide
Myr-3-glu: Myricetin-3*-O*-glucoside
Narin-ch-glu: *Naringenin*-chalcone-4*-O*-glucoside
Quer-3-glr: Quercetin-3*-O*-glucuronide
Quer-3-glu: Quercetin-3*-O*-glucoside
Quer-3-gal: Quercetin-3*-O*-galactoside
Kaempferol-3-glu: Kaempferol-3*-O*-glucoside
Kaempferol-3-glr: Kaempferol-3*-O*-glucuronide
Kaempferol-3-gal: Kaempferol-3*-O*-galactoside
Isorhamnetin-3-glu: Isorhamnetin-3*-O*-glucoside
D-viniferin: delta viniferin
hydroxybenzoic hex: hydroxybenzoic hexoside
Coumaric acid hex: Coumaric acid hexoside
Ferulic acid hex: Ferulic acid hex hexoside
TC: total carotenoid
Chl: Chlorophyll
ROS: reactive oxygen species
Asc: Ascorbate
Hs: accumulated heat stress degree hours
Relx: relaxation degree hours
DDD: daily degree days
MR: Mitzpe Ramon
RN: Ramat Negev
Chenin B: Chenin Blanc
Gewurzt: Gewurztraminer
Muscat A: Muscat of Alexandria
Muscat B: Muscat Blanc
Pinot G: Pinot Gris
Sauvignon B: Sauvignon Blanc.
