# Supplementary material for: The Effect of Topo-Climate Variation on the Secondary Metabolism of Berries in White Grapevine Varieties (Vitis vinifera)
Source: Front Plant Sci. 2022 Mar 8;13:847268. doi: 10.3389/fpls.2022.847268 (PMC8958008; doi:10.3389/fpls.2022.847268)
Supplement: Supplementary file 1 [file Data_Sheet_1.PDF]

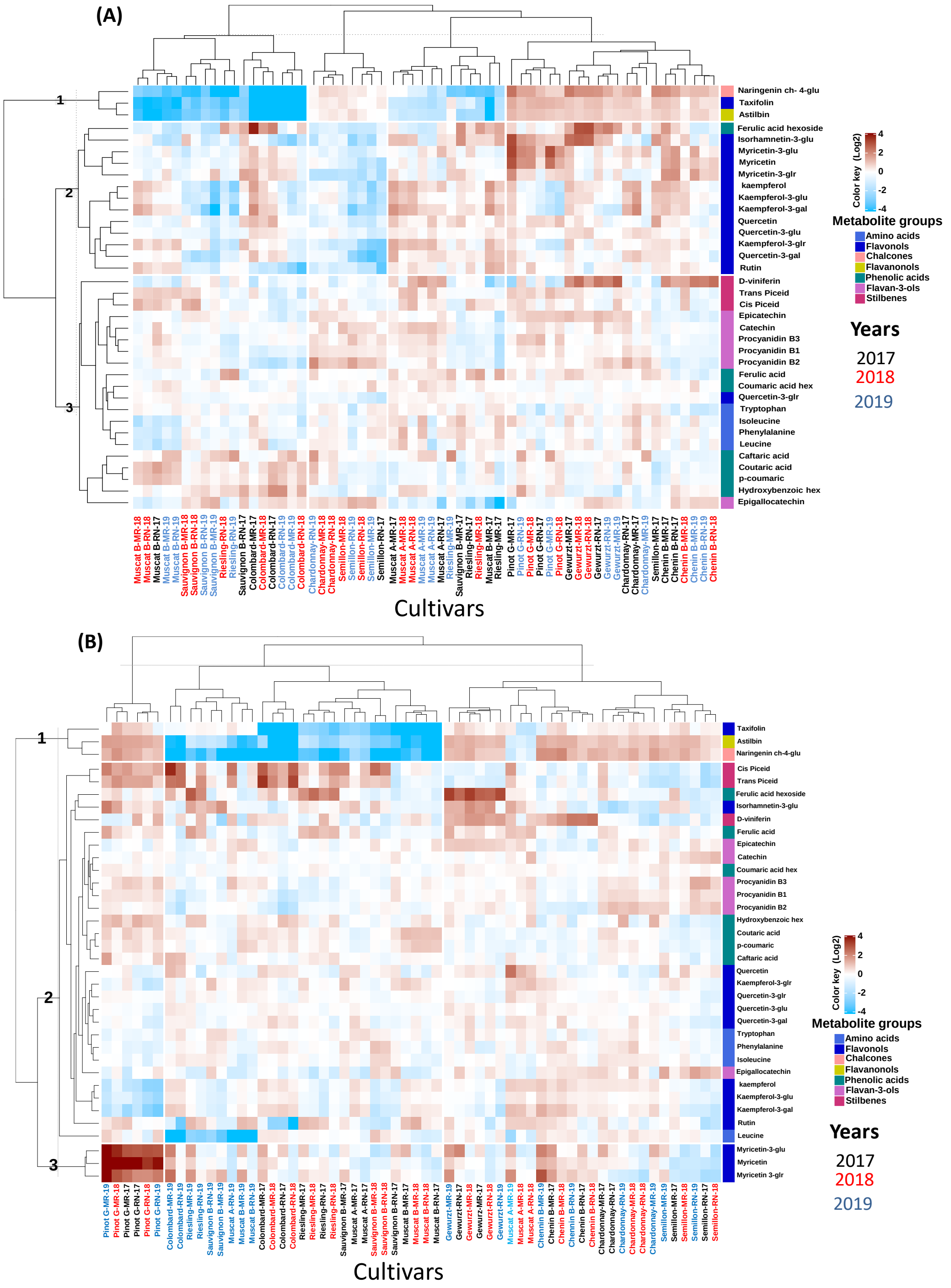

Supplementary *Figure 1:* *Heatmap of skin phenylpropanoids in white grapes at véraison* ***(A)*** *and harvest* ***(B)*** *grown at Mitzpe Ramon (MR) and Ramat Negev (RN) from 2017-2019. The heatmap was generated using the mean value of four biological replicates following normalization to the median of each metabolite on all cultivars and log2 transformation. Cultivar names are denoted by vineyard abbreviations (MR and RN) followed by vintage (17, 18 or 19). Coloured cultivar names indicate samples collected in 2017 (black), 2018 (red), and 2019 (blue). Coloured rectangles represent metabolite increases at MR (red) and RN (blue). Red and blue rectangles represent an increase and decrease of metabolite relative to the median.*

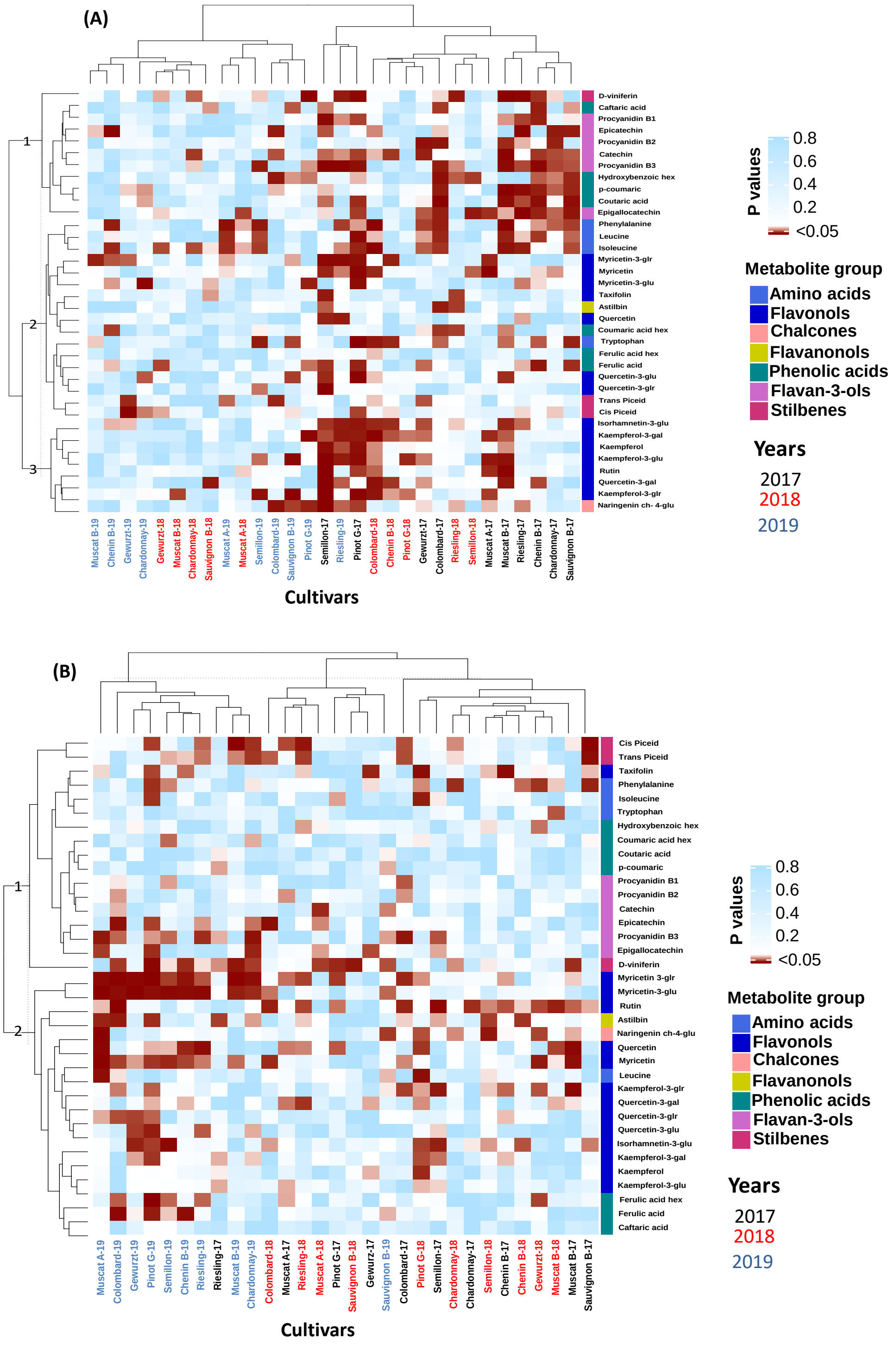

Supplementary Figure 2*: heatmap of skin phenylpropanoids at véraison* ***(A)*** *and harvest* ***(B)*** *that changed significantly between locations during 2017, 2018 and 2019 seasons. Heatmap representation of significant metabolites identified by nonparametric t test between MR and RN. Each metabolite was individually compared between locations for each cultivar in each season. Red coloured rectangles indicate a significant change (P<0.05) of metabolites between locations. Cultivar names are composed by vintage abbreviations (17, 18, 19). Colored cultivar names indicate samples collected in 2017 (black), 2018 (red), and 2019 (blue).*

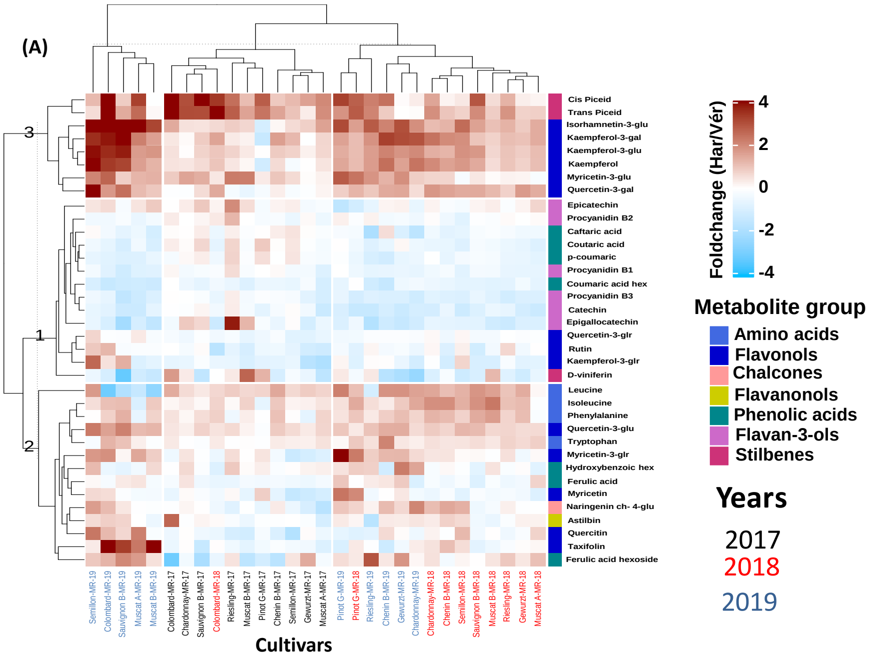

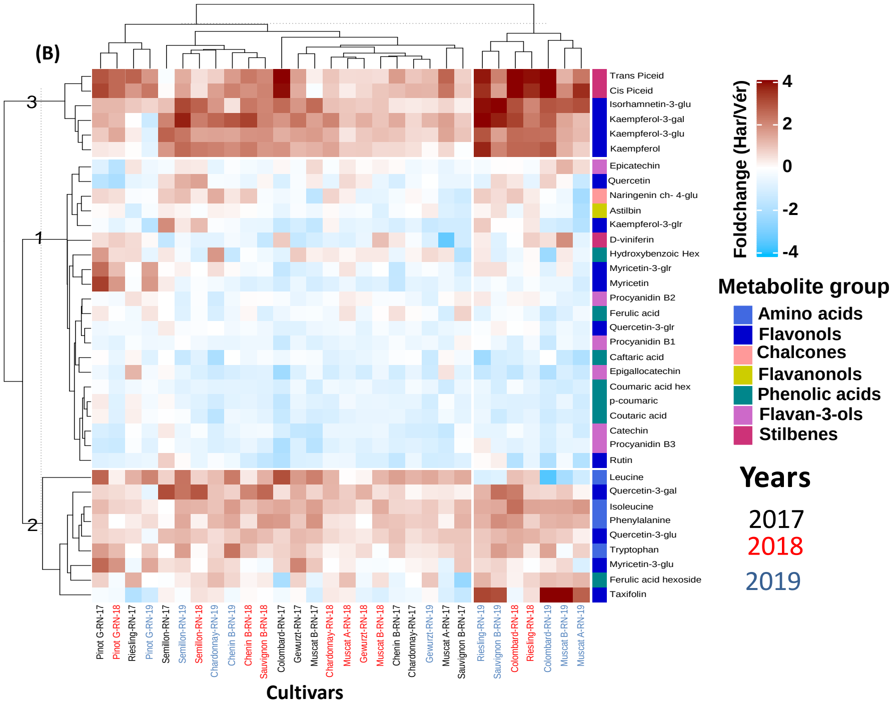

***Supplementary Figure 3**: Change in skin phenylpropanoids from véraison (Vér) to harvest (Har) at Mitzpe Ramon **(A)** and Ramat Negev **(B)** vineyard from 2017-2019, expressed as foldchange (Har/Vér). The mean value of four biological replicates of each metabolite for each cultivar was calculated separately in each season. Then, the values at harvest were divided by the values at véraison. The hierarchical clustering heatmap was generated following log2 transformation. Cultivar names are composed by location abbreviation (MR) and vintage (17, 18, 19). Colored cultivar names indicate samples collected in 2017 (black), 2018 (red), and 2019 (blue). Colored rectangles represent metabolite increases at harvest (red) and véraison (blue).*

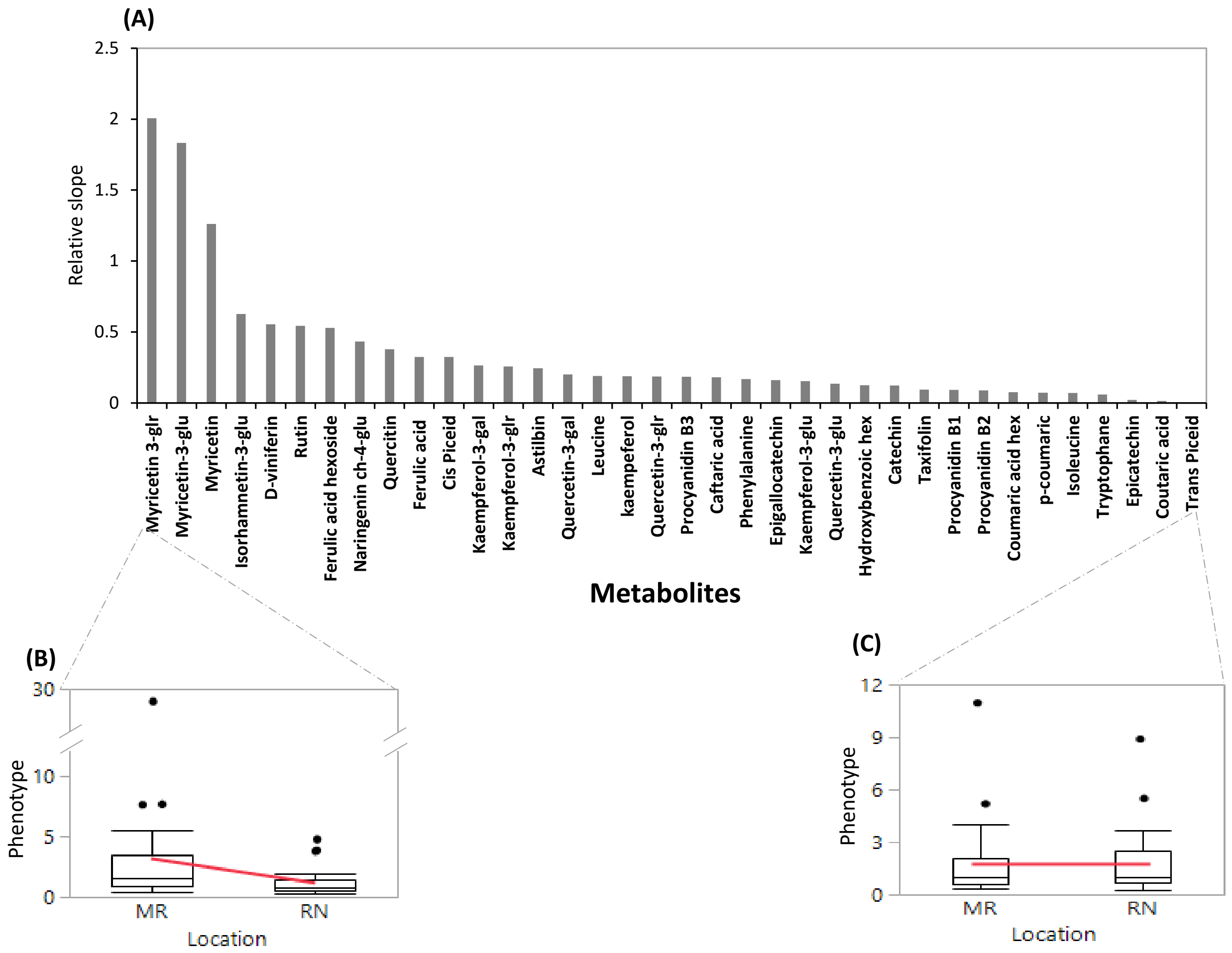

Supplementary Figure 4*: Norm of reaction.* ***(A)*** *Bar graph of the absolute slope values computed for all metabolites.* ***(B)****,* ***(C)*** *Box plots of selected metabolites for Mitzpe Ramon (MR) and Ramat Negev (RN) sites; red lines represent slope. The different metabolites were chosen to demonstrate different genotypic versus environment contributions to the observed cultivar. The slope for metabolites was generated using mean values of each metabolite across all cultivars in each location separately* ***(A)****. Norm of reaction plots was generated following Min-Max data normalization. MR, Mitzpe Ramon; RN, Ramat Negev.*

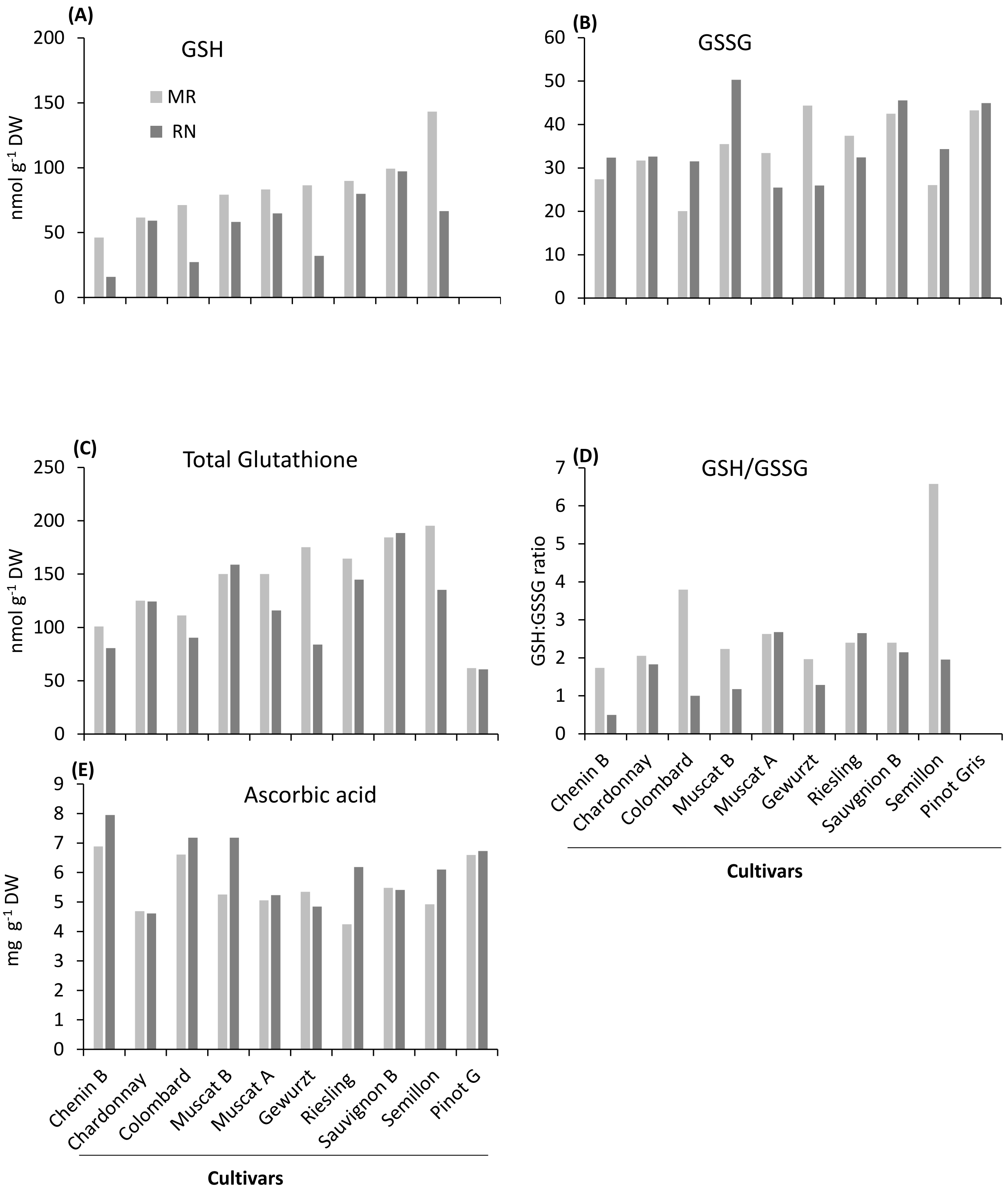

Supplementary Figure 5**: Major redox buffers in the skin of ripe white berries grown at Mitzpe Ramon (MR) and Ramat Negev (RN) in the 2018 season.* ***(A) *Reduced glutathione (GSH),* ***(B)*** *oxidized glutathione (GSSG),* ***(C)*** *total glutathione,* ***(D)*** *GSH/GSSG ratio, and* ***(E)*** *ascorbic acid. Samples were analyzed in bulks (n=bulked replicate x 10 cultivars) at each vineyard location.*
