# Supplementary material for: The Effect of Topo-Climate Variation on the Secondary Metabolism of Berries in White Grapevine Varieties (Vitis vinifera)
Source: Front Plant Sci. 2022 Mar 8;13:847268. doi: 10.3389/fpls.2022.847268 (PMC8958008; doi:10.3389/fpls.2022.847268)
Supplement: Supplementary file 2 [file Data_Sheet_2.docx]

Supplementary Table 1: Total carotenoid content (expressed in µg g^-1^DW) at véraison and harvest in the skin of white cultivar berries grown at Mitzpe Ramon (MR) and Ramat Negev (RN) during 2017-2019 growing seasons.

|  | | **Véraison** | | | | | |  | | |  | **Harvest** | | | | | | | | | |
| --- | --- | --- | --- | --- | --- | --- | --- | --- | --- | --- | --- | --- | --- | --- | --- | --- | --- | --- | --- | --- | --- |
|  |  | **2017** | |  | **2018** | |  | **2019** | |  | | **2017** | |  | **2018** | |  | | **2019** | |  |
|  |  | **MR** | **RN** |  | **MR** | **RN** |  | **MR** | **RN** |  | | **MR** | **RN** |  | **MR** | **RN** | |  | **MR** | **RN** |  |
| **White cultivars** | |  |  | |  |  | |  |  | | |  |  | |  |  | | |  |  | |
|  | Semillon | 43.4+4.1 | **53.2+6.1** | | 87.7+2.5 | 84.1+5.3 | | 75.5+4.0 | 92.9+12.9 | | | 36.0+3.8 | 47.4+3.9 | | **48.4+2.9** | 38.4+5.1 | | | 49.1+0.5 | 40.9+1.4 | |
|  | Muscat A | 51.3+3.1 | **61.4+1.2** | | 62.4+4.5 | 73.6+3.1 | | 63.4+4.1 | 96.3+18.5 | | | 29.9+2.2 | 31.5+1.9 | | 48.2+7.0 | 57.1+3.3 | | | 48.9+3.4 | 49.5+1.6 | |
|  | Riesling | 46.5+3.3 | **65.9+4.8** | | 87.5+3.8 | 89.7+4.9 | | 81.1+1.2 | 113.4+14.6 | | | 47.0+2.5 | 56.2+10.4 | | 53.7+1.6 | 53.6+3.2 | | | 50.2+3.7 | 48.5+2.4 | |
|  | Chenin B | 61.9+2.9 | **83.8+7.7** | | 87.8+2.0 | 92.3+5.0 | | 86.9+1.8 | 85.4+6.8 | | | 45.8+3.8 | 47.8+4.4 | | 53.1+3.1 | 50.7+3.4 | | | 44.9+3.2 | **58.1+3.2** | |
|  | Muscat B | 50.9+3.2 | **86.1+11.4** | | 77.8+8.6 | 66.2+4.8 | | 79.6+8.1 | 73.2+7.1 | | | 39.6+4.4 | 40.1+3.6 | | 53.6+5.5 | 53.2+3.7 | | | 43.7+3.0 | 49.2+3.3 | |
|  | Gewurzt | 62.9+2.3 | **86.3+2.5** | | **78.7+1.3** | 66.9+3.2 | | 90.1+2.4 | 130.8+22.8 | | | 46.0+1.8 | 47.8+1.7 | | **52.5+2.8** | 39.7+2.7 | | | 49.2+2.4 | **59.8+2.9** | |
|  | Colombard | 64.3+2.7 | **92.4+5.1** | | 90.3+4.8 | 90.9+4.5 | | 83.9+2.1 | 114.0+17.4 | | | **42.5+1.8** | 27.2+3.5 | | 57.2+4.3 | 53.1+3.7 | | | 43.7+1.3 | 40.5+1.6 | |
|  | Sauvignon B | 56.1+6.5 | 92.6+6.1 | | 88.9+1.0 | 90.3+3.7 | | 119.7+5.7 | 101.4+6.2 | | | 38.2+1.4 | 48.4+3.8 | | 60.1+1.8 | 51.0+2.6 | | | 41.1+4.7 | **53.4+2.0** | |
|  | Chardonnay | 68.3+7.6 | 98.0+12.4 | | 96.3+6.1 | 76.5+7.2 | | 92.7+4.5 | 126.4+15.8 | | | 52.8+0.9 | 47.7+5.7 | | **60.6+3.5** | 46.3+4.0 | | | 49.2+2.9 | **61.8+1.8** | |
|  | Pinot G | 77.6+7.8 | **107.6+4.9** | | 103.6+0.9 | 104.3+2.6 | | 106.8+6.1 | 86.7+7.6 | | | **53.0+2.6** | 41.9+2.7 | | **74.0+4.5** | 49.1+3.1 | | | 63.8+4.7 | 75.1+1.2 | |
|  | **Two-way ANOVA** | | | | | | | | | | | | | | | | | | | | |
|  | Cultivar (C) | <.0001 | | | <.0001 | | | 0.0004 | | | | <.0001 | | | 0.0023 | | | | <.0001 | | |
|  | Location (L) | <.0001 | | | 0.1754 | | | 0.0039 | | | | 0.9372 | | | 0.0003 | | | | 0.0003 | | |
|  | C x L | 0.5388 | | | 0.0202 | | | 0.0075 | | | | 0.0022 | | | 0.009 | | | | 0.0008 | | |
|  |  |  |  | |  |  | |  |  | | |  |  | |  |  | | |  |  | |
|  | **Three-way ANOVA** | | | | | | | | | | | | | | | | | | | | |
|  | Cultivar | <.0001  <.0001  <.0001  0.3979  <.0001  <.0001  0.0006 | | | | | | | | | | <.0001  0.5288  <.0001  0.0015  <.0001  <.0001  <.0001 | | | | | | | | | |
|  | Location |  |  |  |  |  |  |  |  |  |  |  |  |  |  |  |  |  |  |  |  |
|  | Year (Y) |  |  |  |  |  |  |  |  |  |  |  |  |  |  |  |  |  |  |  |  |
|  | C x L |  |  |  |  |  |  |  |  |  |  |  |  |  |  |  |  |  |  |  |  |
|  | C x Y |  |  |  |  |  |  |  |  |  |  |  |  |  |  |  |  |  |  |  |  |
|  | L x Y |  |  |  |  |  |  |  |  |  |  |  |  |  |  |  |  |  |  |  |  |
|  | C x L x Y |  |  |  |  |  |  |  |  |  |  |  |  |  |  |  |  |  |  |  |  |

Data are means ±SE of four biological replicates. Cultivar means in bold represent significant differences between locations of the same cultivar based on t test.

Supplementary Table 2: Correlation values (R^2^) between total carotenoid degradation (TC), climatic factors (Radiation, DDD, HS and Relx), and H_2_O_2_ in white cultivars grown at Mitzpe Ramon and Ramat Negev vineyards over the period from 2017-2019.

| **Cultivars** | **TC with Radiation** | **TC with DDD** | **TC with HS** | **TC with Relx** | **TC with H_2_O_2_ (R^2^)** |
| --- | --- | --- | --- | --- | --- |
| Chardonnay | 0.02 | 0.24* | 0.14 | 0.07 | 0.04 |
| Chenin Blanc | 0.39** | 0.12 | 0.02 | 0.00 | 0.14 |
| Colombard | 0.46** | 0.41** | 0.44** | 0.56** | 0.02 |
| Gewurztraminer | 0.01 | 0.07 | 0.01 | 0.17* | 0.01 |
| Muscat Alexandria | 0.05 | 0.23* | 0.07 | 0.13 | 0.08 |
| Muscat Blanc | 0.03 | 0.01 | 0.00 | 0.02 | 0.07 |
| Pinot Gris | 0.01 | 0.00 | 0.18* | 0.00 | 0.07 |
| Riesling | 0.19 | 0.30* | 0.02 | 0.05 | 0.05 |
| Sauvignon Blanc | 0.58** | 0.75** | 0.04 | 0.18* | 0.38* |
| Semillon | 0.70** | 0.49** | 0.02 | 0.14 | 0.48* |

The correlations value for each cultivar was generated using four biological replicates (*n*=4 replicates x 2 locations) in the 2017,2018 and 2019 seasons. * indicate a significant correlation. Hs; Heat stress degree hours (Hs), Relx; relaxation degree hours, DDD; daily degree days.

Supplementary Table 3: *P* values of three-way Anova at veraison

| **Metabolite** | **Chemical group** | **Cultivar (C)** | **Location (L)** | **Year (Y)** | **C x L** | **C x Y** | **L x Y** | **C x Lx Y** |
| --- | --- | --- | --- | --- | --- | --- | --- | --- |
| Tryptophane | Amino acids | 0 | 3.57E-14 | 3.07E-08 | 3.90E-06 | 0.000983 | 0.001095 | 7.66E-08 |
| Phenylalanine | Amino acids | 0 | 0 | 4.77E-08 | 0.148564 | 0.006986 | 0.031021 | 4.78E-10 |
| Isoleucine | Amino acids | 0 | 4.77E-15 | 0 | 2.84E-07 | 9.00E-05 | 1.93E-07 | 9.34E-09 |
| Leucine | Amino acids | 0 | 2.00E-15 | 4.09E-11 | 0.001743 | 8.65E-06 | 4.46E-07 | 0.001456 |
| Epigallocatechin | Flavan-3-ols | 0 | 0.001881 | 0.289596 | 5.97E-08 | 5.98E-11 | 3.40E-08 | 0.000204 |
| Catechin | Flavan-3-ols | 0 | 0.064956 | 0.000342 | 0.061872 | 1.39E-09 | 0 | 0.015003 |
| Epicatechin | Flavan-3-ols | 0 | 0.001881 | 0.289596 | 5.97E-08 | 5.98E-11 | 3.40E-08 | 0.000204 |
| Procyanidin B1 | Flavan-3-ols | 0 | 8.73E-06 | 0.016629 | 0.489188 | 0.09904 | 1.98E-05 | 0.122019 |
| Procyanidin B3 | Flavan-3-ols | 0 | 8.66E-10 | 0.010764 | 0.000653 | 0.001569 | 4.07E-13 | 0.000326 |
| Procyanidin B2 | Flavan-3-ols | 0 | 0.005123 | 1.12E-11 | 5.53E-07 | 4.93E-05 | 0.035255 | 0.040825 |
| Astilbin | Flavanonols | 0 | 0.001272 | 3.53E-05 | 0.001248 | 0.000268 | 0.03841 | 0.005248 |
| kaempeferol | Flavanonols | 0 | 2.87E-06 | 0 | 0.022482 | 0.045644 | 0.00127 | 0.040361 |
| Myricetin 3-glr | Flavonols | 0 | 4.26E-14 | 0 | 0.000991 | 0.000263 | 0.000159 | 6.41E-10 |
| Myricetin-3-glu | Flavonols | 0 | 0.001729 | 6.10E-14 | 0.002992 | 0.023664 | 1.66E-05 | 0.001333 |
| Quercetin-3-glr | Flavonols | 0 | 0.000705 | 1.65E-07 | 0.088092 | 0.159067 | 0.006231 | 4.30E-07 |
| Quercetin-3-gal | Flavonols | 0 | 3.57E-07 | 0 | 0.006705 | 0.000207 | 1.74E-07 | 1.70E-06 |
| Quercetin-3-glu | Flavonols | 0 | 5.38E-05 | 2.35E-07 | 0.121719 | 0.000126 | 6.57E-09 | 1.03E-05 |
| Quercitin | Flavonols | 2.22E-16 | 2.53E-05 | 0 | 0.012421 | 5.18E-06 | 0.790875 | 0.000513 |
| Rutin | Flavonols | 0 | 7.98E-12 | 3.39E-09 | 5.06E-06 | 2.31E-10 | 1.13E-08 | 1.03E-13 |
| Kaempferol-3-glr | Flavonols | 0 | 0.000693 | 3.64E-12 | 3.19E-06 | 0.003311 | 4.37E-09 | 6.65E-08 |
| Kaempferol-3-gal | Flavonols | 0 | 2.81E-12 | 0 | 0.029867 | 4.77E-09 | 1.24E-05 | 5.36E-05 |
| Kaempferol-3-glu | Flavonols | 0 | 2.86E-05 | 0 | 6.78E-05 | 1.83E-05 | 1.02E-10 | 2.22E-05 |
| Isorhamnetin-3-glu | Flavonols | 0 | 6.08E-14 | 0 | 0.133997 | 2.71E-10 | 1.84E-11 | 0.000499 |
| Myricetin | Flavonols | 0 | 3.36E-09 | 1.50E-07 | 1.26E-12 | 2.53E-07 | 0.46222 | 8.61E-09 |
| Taxifolin | Flavonols | 0 | 0.000713 | 8.81E-06 | 0.002002 | 0.000853 | 0.600951 | 0.000442 |
| Hydroxybenzoic Hex | Phenolic acids | 0 | 4.38E-10 | 0.000117 | 3.81E-05 | 4.57E-05 | 0.862339 | 4.60E-06 |
| Caftaric acid | Phenolic acids | 0 | 0.014688 | 0 | 0.855915 | 5.55E-16 | 0.009878 | 0.054626 |
| p-coumaric | Phenolic acids | 0 | 0 | 2.94E-10 | 0.004658 | 8.32E-07 | 2.29E-06 | 0.070747 |
| Coutaric acid | Phenolic acids | 0 | 1.43E-14 | 2.42E-06 | 0.006191 | 3.37E-05 | 4.42E-07 | 0.218852 |
| Coumaric acid hex | Phenolic acids | 1.77E-10 | 0.000524 | 1.06E-09 | 0.23504 | 0.296753 | 7.75E-06 | 0.158617 |
| Ferulic acid | Phenolic acids | 0 | 4.21E-07 | 0.707902 | 0.000111 | 5.31E-07 | 1.88E-07 | 0.002814 |
| Ferulic acid hexoside | Phenolic acids | 0 | 0.005857 | 1.04E-14 | 0.401841 | 3.25E-13 | 0.018911 | 0.98533 |
| Naringenin ch-4-glu | Phenylpropanoids | 0 | 0.005845 | 2.98E-12 | 3.70E-06 | 0.00749 | 4.30E-05 | 0.110026 |
| Trans Piceid | Stilbenes | 0 | 0.004685 | 0.001896 | 3.46E-08 | 0.001735 | 0.06467 | 0.045171 |
| Cis Piceid | Stilbenes | 0 | 0.005768 | 1.11E-16 | 0.000193 | 9.10E-11 | 0.00772 | 0.003197 |
| D-viniferin | Stilbenes | 0 | 3.20E-05 | 3.78E-07 | 2.32E-08 | 0.029 | 2.42E-09 | 3.18E-10 |
| Tryptophane | Amino acids | 0 | 3.57E-14 | 3.07E-08 | 3.90E-06 | 0.000983 | 0.001095 | 7.66E-08 |

*P*-values are based on three-way ANOVA taking as main factors: cultivar, Location, and year.

Supplementary Table 4: *P* values of three-way Anova at harvest

| **Metabolite** | **Chemical group** | **Cultivar (C)** | **Location (L)** | **Year (Y)** | **C x L** | **C x Y** | **L x Y** | **C x Lx Y** |
| --- | --- | --- | --- | --- | --- | --- | --- | --- |
| Tryptophane | Amino acids | 0 | 0.033764 | 0.322618 | 0.611326 | 6.66E-16 | 0.00039 | 0.72501 |
| Phenylalanine | Amino acids | 0 | 1.26E-07 | 0.126215 | 0.028493 | 0.000139 | 3.20E-10 | 0.008726 |
| Isoleucine | Amino acids | 0 | 0.02338 | 0.227073 | 0.349338 | 0.000148 | 0.002891 | 0.024622 |
| Leucine | Amino acids | 0 | 4.65E-09 | 0 | 1.78E-05 | 0 | 8.77E-05 | 0.000923 |
| Epigallocatechin | Flavan-3-ols | 0 | 0.009741 | 0.125991 | 5.25E-05 | 0.001004 | 0.036871 | 0.05776 |
| Catechin | Flavan-3-ols | 2.19E-09 | 0.088224 | 0.115652 | 0.005632 | 0.30354 | 0.34781 | 0.429951 |
| Epicatechin | Flavan-3-ols | 0 | 0.592572 | 0.236965 | 0.003597 | 0.000651 | 0.004895 | 0.708861 |
| Procyanidin B1 | Flavan-3-ols | 0 | 0.177419 | 0.465896 | 2.35E-05 | 0.029654 | 0.203726 | 0.873986 |
| Procyanidin B3 | Flavan-3-ols | 0 | 0.000243 | 0.028938 | 8.44E-10 | 0.000517 | 0.000143 | 0.000192 |
| Procyanidin B2 | Flavan-3-ols | 0 | 0.209447 | 0.022353 | 8.20E-08 | 0.170695 | 0.03113 | 0.316632 |
| Astilbin | Flavanonols | 0 | 1.22E-15 | 0.000128 | 9.34E-06 | 2.06E-09 | 0.021699 | 9.70E-10 |
| kaempeferol | Flavanonols | 0 | 8.89E-07 | 0.209936 | 0.043337 | 0.060038 | 0.200449 | 6.20E-05 |
| Myricetin 3-glr | Flavonols | 0 | 0 | 0 | 0.000649 | 1.73E-05 | 0 | 0.00425 |
| Myricetin-3-glu | Flavonols | 0 | 0 | 0 | 0.020529 | 8.53E-06 | 0 | 0.085531 |
| Quercetin-3-glr | Flavonols | 5.33E-11 | 2.99E-10 | 0.000406 | 0.017158 | 0.121164 | 0.736248 | 0.118678 |
| Quercetin-3-gal | Flavonols | 8.88E-15 | 1.63E-06 | 0.244051 | 0.083387 | 0.085792 | 0.000812 | 0.206463 |
| Quercetin-3-glu | Flavonols | 3.50E-12 | 9.46E-08 | 0.680321 | 0.129793 | 0.426768 | 0.000339 | 0.313027 |
| Quercitin | Flavonols | 0 | 2.93E-14 | 0.065915 | 5.13E-10 | 4.05E-11 | 0.000334 | 8.79E-07 |
| Rutin | Flavonols | 0 | 0 | 1.49E-06 | 2.77E-09 | 1.78E-15 | 0.005082 | 1.64E-05 |
| Kaempferol-3-glr | Flavonols | 0 | 7.43E-12 | 0.000299 | 6.89E-05 | 3.05E-07 | 0.146437 | 0.154231 |
| Kaempferol-3-gal | Flavonols | 0 | 3.52E-09 | 0.926341 | 0.00759 | 0.011161 | 0.000376 | 2.94E-06 |
| Kaempferol-3-glu | Flavonols | 0 | 8.99E-08 | 0.25619 | 0.000258 | 0.003706 | 0.010354 | 0.044244 |
| Isorhamnetin-3-glu | Flavonols | 0 | 0 | 3.22E-05 | 5.52E-06 | 7.40E-11 | 0.012605 | 0.006737 |
| Myricetin | Flavonols | 0 | 0 | 2.22E-15 | 1.46E-05 | 6.03E-06 | 2.17E-05 | 0.008999 |
| Taxifolin | Flavonols | 0 | 4.28E-05 | 0 | 0.11357 | 0 | 0.003995 | 0.008857 |
| Hydroxybenzoic Hex | Phenolic acids | 0 | 0.035586 | 0.038688 | 0.9459 | 1.73E-10 | 0.001035 | 0.167283 |
| Caftaric acid | Phenolic acids | 6.26E-08 | 0.000595 | 0.022118 | 0.46699 | 3.45E-07 | 0.003593 | 0.628526 |
| p-coumaric | Phenolic acids | 0 | 0.017708 | 0.000113 | 0.80101 | 0.024259 | 0.191028 | 0.832314 |
| Coutaric acid | Phenolic acids | 0 | 0.514091 | 0.018174 | 0.805157 | 0.014647 | 0.10561 | 0.863303 |
| Coumaric acid hex | Phenolic acids | 6.10E-11 | 0.004891 | 0.891633 | 0.69366 | 0.147565 | 0.055537 | 0.119514 |
| Ferulic acid | Phenolic acids | 0 | 3.30E-11 | 0.987104 | 0.427959 | 7.28E-08 | 1.64E-07 | 0.269085 |
| Ferulic acid hexoside | Phenolic acids | 0 | 0.000499 | 0.163372 | 0.32205 | 0.001065 | 0.293485 | 0.027153 |
| Naringenin ch-4-glu | Phenylpropanoids | 0 | 4.18E-12 | 0.135528 | 5.51E-08 | 2.51E-12 | 0.70899 | 1.12E-08 |
| Trans Piceid | Stilbenes | 0 | 0.55021 | 0.077645 | 2.71E-05 | 2.66E-06 | 7.58E-10 | 1.37E-07 |
| Cis Piceid | Stilbenes | 0 | 0.114964 | 0.014061 | 1.94E-11 | 0 | 4.36E-08 | 1.42E-08 |
| D-viniferin | Stilbenes | 0 | 2.64E-12 | 0.343169 | 2.23E-07 | 9.47E-11 | 3.58E-11 | 1.17E-08 |
| H_2_O_2_ | ROS | <.0001 | <.0001 | <.0001 | 0.0025 | <.0001 | 0.1313 | 0.1376 |

*P*-values are based on three-way ANOVA taking as main factors: cultivar, Location, and year.

Supplementary Table 5: PCA eigen vectors for location sensitive (Muscat of Alexandria), and location and season sensitive (Semillon) cultivars

|  | **Muscat of Alexandria** | | **Semillon** | |
| --- | --- | --- | --- | --- |
| Metabolite | Prin1 | Prin2 | Prin1 | Prin2 |
| Epigallocatechin | 0.18854 | -0.02459 | -0.10853 | 0.30281 |
| Catechin | 0.03439 | 0.22769 | -0.18554 | 0.09719 |
| Epicatechin | 0.09015 | 0.07434 | -0.16183 | 0.2876 |
| Hydroxybenzoic Hex | 0.08881 | 0.07185 | 0.08654 | 0.19525 |
| Caftaric acid | 0.15655 | 0.00013 | 0.1419 | 0.09399 |
| Procyanidin B1 | 0.00566 | 0.22317 | -0.19105 | 0.20814 |
| Procyanidin B3 | -0.09079 | 0.25568 | -0.20514 | 0.16513 |
| Procyanidin B2 | -0.0491 | 0.12056 | -0.17891 | 0.14999 |
| p-coumaric | 0.05185 | 0.09885 | -0.04174 | -0.10224 |
| Coutaric acid | 0.13368 | 0.21064 | -0.04646 | 0.14235 |
| Coumaric acid hex | 0.10208 | 0.19272 | 0.02607 | 0.09765 |
| Ferulic acid | 0.21939 | 0.10184 | 0.15043 | -0.07297 |
| Myricetin 3-glr | 0.23794 | 0.11316 | 0.16353 | 0.25145 |
| Myricetin-3-glu | 0.18426 | 0.06267 | 0.13431 | 0.14493 |
| Quercetin-3-glr | 0.24498 | 0.08679 | 0.1218 | 0.22313 |
| Quercetin-3-gal | 0.14345 | -0.11408 | 0.24037 | 0.08096 |
| Quercetin-3-glu | 0.13361 | -0.11941 | 0.23952 | 0.06471 |
| Quercitin | 0.24675 | 0.1032 | 0.11281 | 0.17213 |
| Rutin | 0.25931 | 0.02417 | 0.23161 | 0.06479 |
| Kaempferol-3-glr | 0.20904 | 0.08614 | 0.27353 | -0.00967 |
| Kaempferol-3-gal | 0.24625 | -0.1332 | 0.21652 | -0.08884 |
| Kaempferol-3-glu | 0.20203 | -0.20999 | 0.25599 | -0.07598 |
| Naringenin ch-4-glu | 0.19795 | -0.0775 | 0.22256 | 0.1319 |
| Trans Piceid | -0.10679 | 0.20738 | 0.06828 | 0.15781 |
| Cis Piceid | -0.06996 | 0.27136 | 0.05269 | 0.21429 |
| D-viniferin | 0.05405 | 0.30293 | -0.11515 | 0.25036 |
| Astilbin | 0.25603 | -0.07521 | 0.16359 | 0.14505 |
| Isorhamnetin-3-glu | 0.23927 | -0.03913 | 0.27227 | -0.01304 |
| Ferulic acid hexoside | 0.18205 | -0.03301 | 0.11257 | -0.18615 |
| Myricetin | 0.26332 | 0.06972 | 0.16931 | 0.24492 |
| Taxifolin | 0.03989 | 0.30904 | 0.13591 | -0.2149 |
| Tryptophane | -0.12502 | -0.19231 | 0.10918 | -0.19484 |
| Phenylalanine | 0.07942 | -0.24471 | 0.08511 | -0.01863 |
| Isoleucine | -0.03327 | -0.21273 | 0.14551 | -0.01959 |
| Leucine | 0.04446 | -0.31979 | 0.10984 | 0.27376 |
| kaempeferol | 0.24569 | -0.07768 | 0.22467 | 0.10774 |

Supplementary Table 6: Summary of Mitzpe Ramon and Ramat Negev correlation networks that were constructed using UPLC-QTof-MS generated metabolite profiles at harvest and climate indices during ripening.

| Network summary | Locations | |
| --- | --- | --- |
|  | Ramat Negev | Mizpe Ramon |
| Number of nodes | 44 | 44 |
| Number of edges | 194 | 180 |
| Average number of neighbors | 8.818 | 8.182 |
| Network diameter | 4 | 5 |
| Network radius | 3 | 3 |
| Characteristics path length | 2.167 | 2.174 |
| Clustering coefficient | 0.495 | 0.385 |
| Network density | 0.205 | 0.19 |
| Network heterogeneity | 0.567 | 0.475 |
| Network centralization | 0.248 | 0.215 |
